# Cardiomyocyte proliferation is suppressed by ARID1A-mediated YAP inhibition during cardiac maturation

**DOI:** 10.1038/s41467-023-40203-2

**Published:** 2023-08-05

**Authors:** Cornelis J. Boogerd, Ilaria Perini, Eirini Kyriakopoulou, Su Ji Han, Phit La, Britt van der Swaan, Jari B. Berkhout, Danielle Versteeg, Jantine Monshouwer-Kloots, Eva van Rooij

**Affiliations:** 1 Hubrecht Institute, Royal Netherlands Academy of Arts and Sciences (KNAW) and University Medical Center Utrecht, Utrecht, Netherlands; 2 Department of Cardiology, University Medical Center Utrecht, Utrecht, Netherlands

**Keywords:** Cardiac regeneration, Heart development, Cell proliferation

## Abstract

The inability of adult human cardiomyocytes to proliferate is an obstacle to efficient cardiac regeneration after injury. Understanding the mechanisms that drive postnatal cardiomyocytes to switch to a non-regenerative state is therefore of great significance. Here we show that *Arid1a*, a subunit of the switching defective/sucrose non-fermenting (SWI/SNF) chromatin remodeling complex, suppresses postnatal cardiomyocyte proliferation while enhancing maturation. Genome-wide transcriptome and epigenome analyses revealed that *Arid1a* is required for the activation of a cardiomyocyte maturation gene program by promoting DNA access to transcription factors that drive cardiomyocyte maturation. Furthermore, we show that ARID1A directly binds and inhibits the proliferation-promoting transcriptional coactivators YAP and TAZ, indicating ARID1A sequesters YAP/TAZ from their DNA-binding partner TEAD. In ischemic heart disease, *Arid1a* expression is enhanced in cardiomyocytes of the border zone region. Inactivation of *Arid1a* after ischemic injury enhanced proliferation of border zone cardiomyocytes. Our study illuminates the pivotal role of *Arid1a* in cardiomyocyte maturation, and uncovers *Arid1a* as a crucial suppressor of cardiomyocyte proliferation.

## Abstract (web summary)

Cardiac regeneration is hindered by the limited division of cardiomyocytes. Here, the authors show that Arid1a drives maturation and limits proliferation through interaction with Yap. Suppression of Arid1a enhances proliferation after injury.

## Introduction

During postnatal development, mammalian cardiomyocytes switch from a pro-regenerative, proliferative state towards mature, non-regenerative cells equipped for a lifetime of forceful contractions^1,2^. Diminished proliferative capacity of adult cardiomyocytes forms a barrier to replenish the massive loss of cardiomyocytes after ischemic injury. To date, no efficient treatment to regenerate cardiomyocytes is available, highlighting the need for mechanistic understanding of the processes regulating the balance between cardiomyocyte proliferation and maturation. Natural cardiomyocyte regeneration occurs through the proliferation of existing cardiomyocytes^3^. Cardiomyocyte proliferation capacity is quickly lost after birth, concurrent with adaptations characteristic of cardiomyocyte maturation^4–6^. Factors that induce cardiomyocyte maturation, such as oxygen and thyroid hormone, have a suppressive effect on cardiomyocyte proliferation^7–9^. Adult cardiomyocyte proliferation can be triggered by overexpression of active yes associated protein 1 (YAP), a Hippo pathway effector that controls organ size^10,11^. In the neonatal heart, Hippo pathway kinases suppress YAP, thereby limiting cardiomyocyte proliferation^12,13^. Forced activation of YAP in postnatal cardiomyocytes induces proliferation, enlarged hearts, and decreased maturation^14,15^. In adult cardiomyocytes, ectopic YAP activaty causes cardiac overgrowth, impairing heart function and ultimately causing lethality^10,16^.

Furthermore, cardiomyocyte proliferation and maturation are important for tissue engineering approaches. Despite recent advances promoting stem cell-derived cardiomyocyte maturation, complete maturation of induced pluripotent stem cell-derived cardiomyocytes (iPS-CMs) has yet to be achieved^17,18^. Incomplete maturation of iPS-CMs and in vitro engineered tissue poses a major limitation in leveraging their potential for in vitro modeling for pathological, pharmacological, or therapeutic purposes. Therefore, insights into the molecular underpinnings of cardiomyocyte maturation are imperative to further enhance in vitro tissue maturation and the advancement of cardiac regenerative medicine.

Loss of regenerative potential during postnatal cardiac maturation coincides with dramatic changes to the epigenetic landscape. Through chromatin remodeling, DNA methylation, and histone modifications postnatal cardiomyocytes achieve stable silencing of cell cycle genes, and activation of structural, metabolic, and electrophysiological maturation gene programs^19–21^. Despite recent advances, our understanding of epigenetic mechanisms involved in cardiac maturation remains incomplete^17,19,22^.

The SWI/SNF complex is a multiprotein chromatin remodeling complex with essential functions during cardiac lineage specification and cardiomyocyte differentiation^20,23,24^. In the adult heart, the SWI/SNF complex is involved in stress-induced re-expression of fetal genes and cardiomyocyte hypertrophic growth^25^. SWI/SNF complex modulation of chromatin accessibility at target genes is facilitated by AT-rich interactive domain-containing protein 1a (ARID1A)^26^. *Arid1a* has essential roles in controlling cell proliferation^27^, and suppresses tissue regeneration in the context of liver or outer ear injury^28^. Despite essential functions during cardiac specification and differentiation^29–32^, roles of *Arid1a* in cardiac maturation and adult heart function so far remained unexplored.

Here, we describe roles of *Arid1a* during mammalian postnatal cardiomyocyte maturation. We show that *Arid1a* is expressed in neonatal mouse cardiomyocytes and that its expression is enhanced in adult cardiomyocytes after ischemic injury. Loss of *Arid1a* in neonatal mouse cardiomyocytes, as well as human iPS-CMs, leads to increased proliferation and suppression of cardiac maturation gene programs. Furthermore, overexpression of ARID1A enhanced functional maturation of engineered human myocardium (EHM). Combined ChIP-Seq analysis and motif discovery revealed changes in epigenetic marks and induction of YAP dependent gene programs, suggesting ARID1A suppresses YAP activity in neonatal hearts. We show that ARID1A directly interacted with YAP and suppressed its transcriptional activity. Similarly, in adult cardiomyocytes, suppression of ARID1A enhanced proliferation in border zone cardiomyocytes after ischemic injury. Together, our results identify *Arid1a* as an essential regulator of the cardiomyocyte proliferation to maturation switch, providing novel inroads for development of cardiac regenerative applications.

## Results

### Cardiomyocytes express *Arid1a* after birth and after injury

**Fig. 1:**
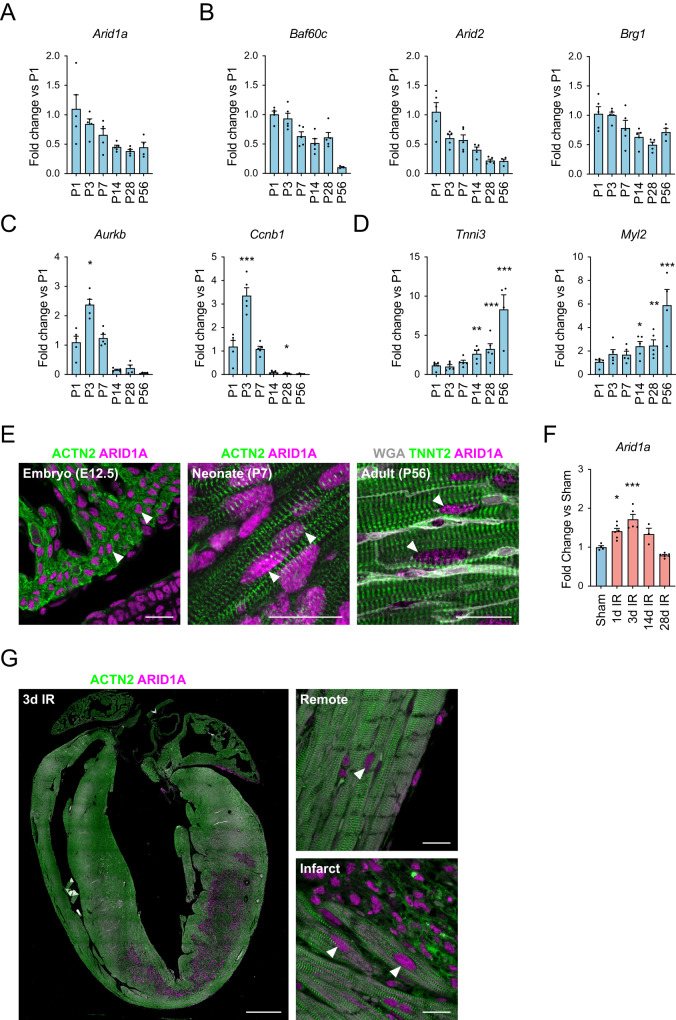
*Arid1a* expression during postnatal heart maturation. **A**–**C** Real-time quantitative PCR (qPCR) determined levels of gene expression during postnatal cardiac development. Levels are shown as fold change over P1 for (**A**) *Arid1a*, (P1-P28: *n* = 5, P56: *n* = 4) (**B**) other SWI/SNF complex subunits *Baf60c* (P1-P28: *n* = 5, P56: *n* = 3), *Arid2* (P1-P28: *n* = 5, P56: *n* = 4) and *Brg1* (P1-P28: *n* = 5, P56: *n* = 4), (**C**) proliferation markers *Aurkb* (P1-P14: *n* = 5, P28,P56: *n* = 4; P3 *P* = 0.0214) and *Ccnb1* (P1-P28: *n* = 5, P56: *n* = 4; P3 *P* < 0.0001, P28 *P* = 0.0498), and (**D**) cardiomyocyte maturation markers *Tnni3* (P1-P28: *n* = 5, P56: *n* = 4; P14 *P* = 0.0043, P28 *P* < 0.0001; P56 *P* < 0.0001) and *Myl2* (P1-P28: *n* = 5, P56: *n* = 4; P14 *P* = 0.0156, P28 *P* = 0.0096, P56 *P* < 0.0001). **E** Representative example of immunofluorescence staining for ARID1A (red) in embryonic (E12.5; *n* = 3), postnatal (P7; *n* = 4) and adult (8-10w; *n* = 4) mouse cardiomyocytes (ACNT2 or TNNT2, green). Extracellular matrix is highlighted by WGA staining in the adult section. **F** *Arid1a* expression (qPCR) in sham (*n* = 4) and at 1 (*n* = 6; *P* = 0.0099), 3 (*n* = 5; *P* < 0.0001), 14 (*n* = 3) and 28 (*n* = 6) days (d) after ischemia-reperfusion injury (IR). **G** ARID1A expression in mouse heart 3 days post IR (*n* = 5). Arrowheads mark ARID1A positive cardiomyocyte nuclei near infarct area. Scale bars 1 mm (**G**, left panel), 20 µm (**E**; **G** right panels). Bar graphs show mean and standard error of the mean; one-way ANOVA with Dunnett’s test for multiple comparisons, compared to P1 (**A**–**D**) or Sham (**F**): *P < 0.05; **P < 0.01; ***P < 0.001. Source data are provided as a Source Data file.

The mammalian heart switches from a pro-regenerative, proliferative state towards a mature, non-regenerative state during the first days after birth^1^. We hypothesized that *Arid1a*, a known regulator of the balance between proliferation and differentiation, and a subunit of the SWI/SNF chromatin remodeling complex^27^, may be involved in this process in postnatal cardiomyocytes. To examine this, we first evaluated *Arid1a* expression in the heart using qPCR and found that *Arid1a* was expressed abundantly in neonatal hearts and decreases toward adulthood (Fig. 1A). Similarly, other SWI/SNF chromatin remodeling complex genes were expressed highly in the newborn heart, with levels decreasing with age (Fig. 1B)^25,33^. The transient activation of cell cycle activity in cardiomyocytes after birth^6^ was evident by a short spike in Aurora B kinase (*Aurkb*) and Cyclin b1 (*Ccnb1*) expression (Fig. 1C), while a gradual increase in the sarcomeric proteins Troponin I (*Tnni3*) and myosin light chain 2 (*Myl2*) exemplified contractile protein isoform switch typical of cardiomyocyte maturation (Fig. 1D).

To further assess its spatiotemporal expression pattern, we performed immunostainings and detected ARID1A in cardiomyocytes during development, and in neonatal hearts. ARID1A was also detected in adult cardiomyocytes (Fig. 1E, Supplemental Fig. 1A). In mouse hearts subjected to ischemia-reperfusion injury (IR), *Arid1a* expression was induced 1 and 3 days after injury (Fig. 1F). Next, we used immunofluorescence imaging to determine the cell types contributing to this increase. In addition to many ARID1A positive cells in the scar area, which is rich in immune cells and fibroblasts, we also noted that ARID1A was enhanced in cardiomyocytes bordering the infarct zone (Fig. 1G). Upregulation of ARID1A in border zone cardiomyocytes could further be confirmed in a mouse model of myocardial infarction (MI; Supplemental Fig. 1B, C).

**Fig. 2:**
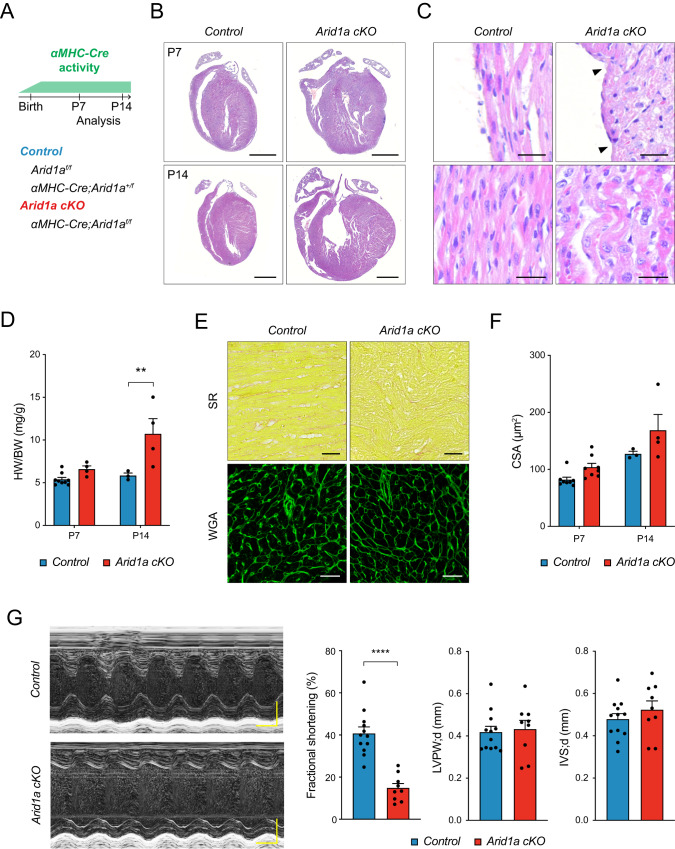
*Arid1a* is required in neonatal cardiomyocytes for heart maturation. **A** Schematic showing analysis timeline for cardiomyocyte specific *Arid1a* mutants (*Arid1a cKO*). **B** Representative images of hematoxylin and eosin (H&E) staining of P7 and P14 control and *Arid1a cKO* hearts. **C** Uneven surface (arrowheads) and signs of myocardial disarray (bottom) in *Arid1a cKO* hearts (*n* = 4) compared to controls (*n* = 5) at P14. **D** Heart weight to body weight ratio (HW/BW) at P7 (Control, *n* = 9; *Arid1a cKO*, *n* = 4) and P14 (Control, *n* = 3; *Arid1a cKO*, *n* = 4; *P* = 0.0029; **P < 0.01, two-way ANOVA with Šídák’s multiple comparisons test). **E** Sirius red (SR) staining for fibrosis and wheat germ agglutinin (WGA) staining for extracellular matrix. **F** Quantification of cardiomyocyte cross-sectional area (CSA) as measured from WGA-stained hearts at P7 (*n* = 8) and P14 (Control, *n* = 3; *Arid1a cKO*, *n* = 4) revealed no significant difference between *Arid1a cKO* and control at P7 (*P* = 0.2049) or P14 (*P* = 0.1021; two-way ANOVA with Šídák’s multiple comparisons test). **G** Representative example of echocardiography analysis of cardiac function in P7 hearts, with quantification of fractional shortening (*P* < 0.0001), interventricular septum thickness (IVS; *P* = 0.7787) and left ventricular peripheral wall thickness (LVPW; *P* = 0.3624) during diastole (d) (Control, *n* = 12; *Arid1a cKO*, *n* = 9; ****P < 0.0001, two-tailed Student’s *t*-test). Scale bars 1 mm (**B**), 25 µm (**C**), 50 µm (**E**), 0,1 s (**G**, horizontal), 0,1 mm (**G**, vertical). All bar graphs show mean with standard error of the mean. Source data are provided as a Source Data file.

Next, we set out to characterize *Arid1a* function in neonatal cardiomyocytes. As *Arid1a* is required in multiple cell lineages during cardiogenesis^29,34,35^, we utilized the cardiomyocyte-specific alpha myosin heavy chain *Cre* (*αMHC-Cre*)^36^ to ablate *Arid1a* (Fig. 2A). Using qPCR, immunostaining, and *αMHC-Cre*;*Rosa26-tdTomato* lineage tracing, we validated efficient *Arid1a* deletion from cardiomyocytes at postnatal day 7 (P7) in *αMHC-Cre;Arid1a* mutants (*Arid1a cKO*; Supplemental Fig. 2A–D). At P1, the majority of cardiomyocytes still expressed high levels of ARID1A, in line with robust perinatal activation of the *αMHC-Cre* transgene (Supplemental Fig. 2B,D)^36^.

Histological examination at P7 and P14 revealed that *Arid1a* was required for normal heart maturation or function. At P7, *Arid1a cKO* hearts were malformed, as marked by local indentations and bulges at the outer ventricular surface (Fig. 2B). This was even more pronounced at P14, and mutant hearts were dramatically enlarged, with severe myocardial disarray (Fig. 2B, C). Average heart weight (HW) had doubled by P14 (Fig. 2D), with no evidence of increased fibrosis (Fig. 2E). Although a modest increase in average cross-sectional area (CSA) was noted at P14, it was not statistically significant, indicating that cardiomyocyte hypertrophy was unlikely to account for increased mutant heart sizes at this stage (Fig. 2E, F).

Next, we evaluated cardiac function in *Arid1a cKO* and control littermates at P7 using echocardiography and found that loss of *Arid1a* resulted in decreased left ventricular systolic function (fractional shortening; Fig. 2G). In line with histological data, diastolic interventricular septum (IVS) and left ventricular peripheral wall (LVPW) thickness were not changed at this stage (Fig. 2G). Together, these data reveal essential roles of *Arid1a* in cardiomyocytes during postnatal maturation.

### *Arid1a* promotes maturation and suppresses proliferation

**Fig. 3:**
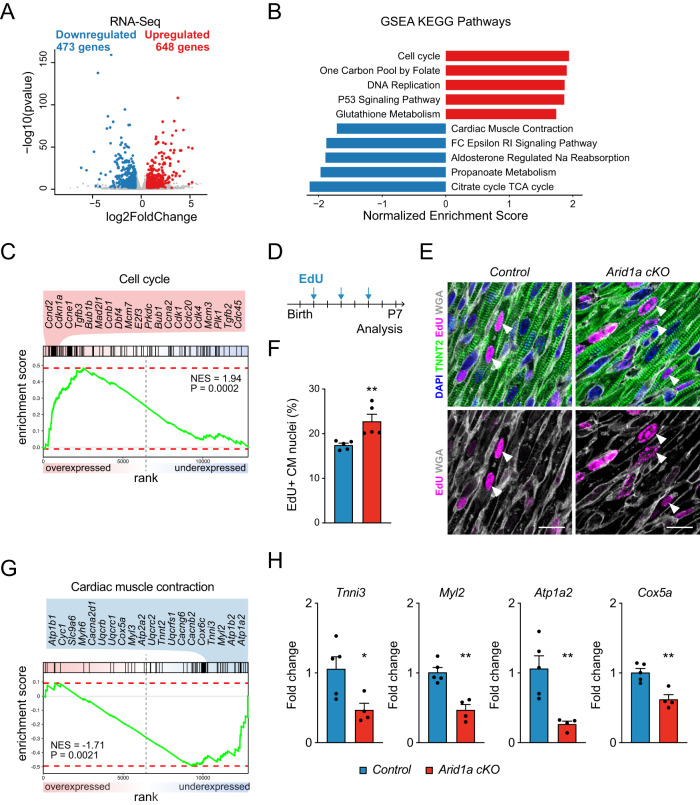
Increased proliferation and decreased maturation in *Arid1a cKO* cardiomyocytes. **A** Volcano plot of RNA-Seq on P7 hearts with 473 genes downregulated (blue) and 648 genes upregulated (red) in *Arid1a cKO* (*n* = 4) compared to control (*n* = 5) hearts (Differentially expressed genes are defined as those genes with an absolute fold change >1.5x and Wald-test with Benjamini-Hochberg-adjusted *P* < 0.05). **B** Gene set enrichment analysis (GSEA) for Kyoto encyclopedia of genes and genomes (KEGG) identified pathways activated (red) or suppressed (blue) in *Arid1a cKO* compared to control hearts. **C** GSEA enrichment plot with most significantly induced *cell cycle* genes in *Arid1a cKO* hearts. **D** Schematic showing EdU incorporation assay timeline. EdU was administered to neonates at P1, P3 and P5. Hearts were analyzed at P7. **E** Representative example of EdU staining (magenta) in P7 *Arid1a cKO* and control hearts, co-stained with TNNT2 (green) and WGA (gray) to identify cardiomyocytes. Scale bars 20 µm. **F** Quantification of EdU incorporation in P7 control and *Arid1a cKO* hearts (*n* = 5; *P* = 0.0079). **P < 0.01, two-tailed Mann–Whitney test. **G** GSEA enrichment plot with most significantly suppressed *cardiac muscle contraction* genes in *Arid1a cKO* hearts. **H** Levels of mRNA transcripts encoding sarcomeric proteins (*Myl2*, *P* = 0.0012; *Tnni3*, *P* = 0.0269, ion channels (*Atp1a2*, *P* = 0.0066) and energy metabolism (*Cox5a*, *P* = 0.0032) in *Arid1a cKO* (*n* = 4) and control hearts (*n* = 5) as determined by qPCR). *P < 0.05; **P < 0.01, two-tailed Student’s *t*-test. All bar graphs show mean with standard error of the mean. Source data are provided as a Source Data file.

To assess the underlying transcriptional characteristics of *Arid1a* mutant hearts we performed RNA-Seq and compared *Arid1a cKO* and littermate control hearts at P7. We identified marked changes in gene expression, with 473 downregulated and 648 upregulated genes (Fig. 3A). RNA-Seq confirmed a marked downregulation of *Arid1a* transcripts, while its homologs *Arid1b* and *Arid2* were not changed, indicating the absence of a compensatory mechanism (Supplemental Fig. 2E). Consistent with the observed enlargement of mutant hearts, genes involved in cell cycle and DNA synthesis were significantly upregulated in mutant hearts (Fig. 3B; Supplementary Data 1). Among the most significantly overexpressed cell cycle genes were critical cardiomyocyte cell cycle regulators, such as cyclin D2 (*Ccnd2*), *Ccne1*, *Ccnb1*, *Ccna2*, cyclin-dependent kinase 1 (*Cdk1*), and *Cdk4* (Fig. 3C)^6^. Also, the E2F transcription factor 3 (E2F3), which is required for cardiomyocyte proliferation, was upregulated in *Arid1a cKO* hearts^37^. To evaluate whether increased expression of cell cycle genes translated into increased cardiomyocyte proliferation, we injected the thymidine analog EdU (5-ethynyl-2´-deoxyuridine) subcutaneously in pups at P1, P3, and P5 and assessed EdU incorporation at P7 (Fig. 3D). In control hearts, 17.5% of cardiomyocytes had undergone S-phase, whereas this number was significantly increased in mutant hearts (22.4%; Mann–Whitney *P* = 0.0079; Fig. 3E, F).

Downregulated genes were associated with characteristics of cardiomyocyte maturation, such as cardiac muscle contraction and energy metabolism (Fig. 3B, G; Supplementary Data 1). For example, the expression of *Myh6*, which encodes αMHC and is the predominant MHC isoform in adult mouse cardiomyocytes, was downregulated in *Arid1a cKO* hearts (Fig. 3G). Similarly, cardiac maturation markers including *Tnni3* and *Myl2* were downregulated in *Arid1a cKO* mutants (Fig. 3G, H). In addition, calcium handling proteins, which normally increase with cardiac maturation^2^, were downregulated in *Arid1a cKO* hearts. Key downregulated genes in this pathway included the ATPase Sarcoplasmic/Endoplasmic Reticulum Ca^2+^ Transporting 2 (SERCA, encoded by *Atp2a2*) and multiple homologous ion-transporting ATPases (*Atp1a2*, *Atp1b2* and *Atp1b1*), as well as voltage-dependent calcium channels *Cacna2d1*, *Cacnb2*, and *Cacng6*. Finally, we observed decreased energy metabolism gene expression in mutants, including Cytochrome C Oxidase Subunit 5 A (*Cox5a*) and *Cox6c* (Fig. 3G, H). Taken together, these results demonstrate that *Arid1a* is required for cell cycle withdrawal during postnatal cardiac maturation, and regulates multiple aspects of cardiomyocyte maturation, including the expression of genes related to contraction, energy metabolism, and calcium handling.

### *ARID1A* function is conserved in human cardiomyocytes

To understand whether functions of ARID1A in cardiomyocyte maturation were conserved between mouse and human, we used siRNA and suppressed ARID1A in human iPS-derived cardiomyocytes (iPS-CMs; Supplemental Fig. 3A,B). First, we performed EdU incorporation assays and found that suppression of ARID1A led to a significant increase in proliferation, providing further evidence that ARID1A suppresses cardiomyocyte proliferation (Supplemental Fig. 3C). Furthermore, qPCR analysis further confirmed that ARID1A promotes cardiomyocyte maturation genes, including contractility (Supplemental Fig. 3D; *TNNI3*, *MYL2*, *MYH7*) and ion homeostasis genes (Supplemental Fig. 3E; *PLN*, *ATP2A2*, *RYR2*, *GJA1*). However, metabolism genes that were downregulated in *Arid1a* cKO mice were not changed to the same extent in human iPS-CMs (Supplemental Fig. 3F; *COX5A*, *COX6C*, *CPT1B*). Decreased expression of *PLN* and *TNNI3* translated into a trend towards a similar decrease at the protein level (Supplemental Fig. 3G).

**Fig. 4:**
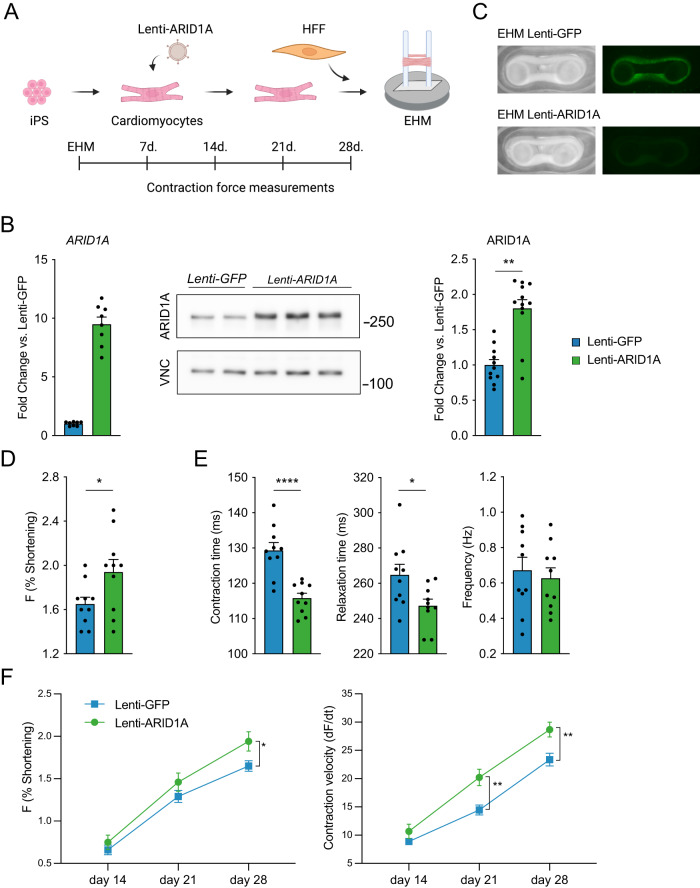
ARID1A overexpression in cardiomyocytes enhances maturation of engineered human myocardium. **A** Schematic overview of cardiomyocyte differentiation and transduction with Lenti-ARID1A (or Lenti-GFP as control) and casting with human foreskin fibroblasts (HFF) to generate engineered human myocardium (EHM). Contraction force and kinetics measurements were performed weekly for 4 weeks. **B** Quantification of *ARID1A* RNA by qPCR (left, *n* = 2 independent differentiations, with 4 technical replicates each), western blot (middle) and quantification of ARID1A protein level (right; *P* = 0.0022, two-tailed Student’s *t*-test on average of 4 independent differentiations; graph shows 4 independent differentiations, with 2 or 3 replicates each); (**P < 0,01, two-tailed Student’s *t*-test) in iPS-CM transduced with Lenti-ARID1A or Lenti-GFP. Molecular weight marker (kDa) is shown at the right. **C** Representative images of EHM tissue (left) and GFP fluorescence (right) at the end of the experiment showing sustained expression of the Lentiviral cargo. **D**–**F** EHM contraction measurements, *n* = 10 engineered human myocardium rings from a single experiment; *P < 0.05, **P < 0.01, ****P < 0.0001, two-tailed Student’s *t*-test, performed separately for each timepoint. **D** Force of contraction (**F**; *P* = 0.0380) measurements of Lenti-GFP or Lenti-ARID1A EHM at 4 weeks after casting**. E** Contraction time(P < 0.0001), relaxation time (*P* = 0.0225), and contraction frequency (*P* = 0.6375) at 4 weeks (*n* = 10; *P < 0.05, two-tailed Student’s *t*-test). **F** Force of contraction (left) and contraction velocity (dF/dt) measurements over time. Graphs show mean with standard error of the mean. Source data are provided as a Source Data file. **A** Created with BioRender.com.

Next, we utilized engineered human myocardium (EHM), which offers more advanced and mature cardiomyocytes with functional readouts of contractility^18,38^. We hypothesized that overexpression of ARID1A might be sufficient to promote cardiomyocyte maturation. EHMs were cast from iPS-CMs transduced with Lenti-GFP or Lenti-ARID1A (Fig. 4A, B), and contractility measurements were performed weekly for 4 weeks (Fig. 4A, C). After 4 weeks, ARID1A overexpression had induced an increase in the force of contraction (F, % shortening, Fig. 4D, F). Moreover, contraction time and relaxation time were significantly decreased after ARID1A overexpression, while contraction frequency was not significantly changed (Fig. 4E). Contraction velocity was also increased after 3 weeks and 4 weeks, further indicating that contraction kinetics were more mature (Fig. 4F). Collectively, our findings in neonatal mouse hearts, human iPS-CMs and human EHMs indicate an essential, conserved role for Arid1a in enhancing cardiomyocyte maturity at the expense of proliferation.

### Loss of *Arid1a* modifies the cardiac chromatin landscape

**Fig. 5:**
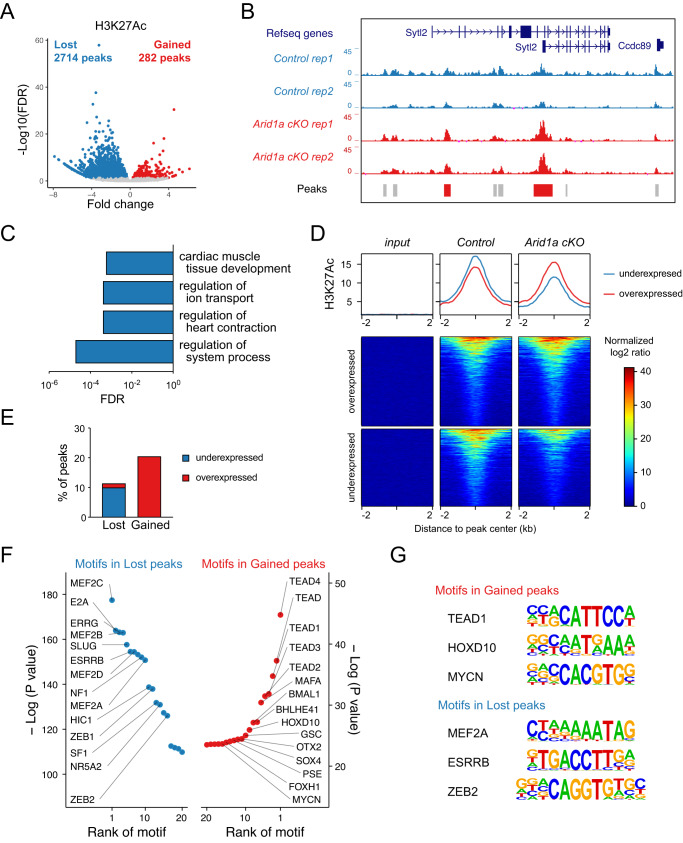
Loss of *Arid1a* changes the chromatin landscape in cardiac maturation. **A** Volcano plot of H3K27Ac ChIP-Seq on P7 hearts identifies 282 peaks with gained H3K27Ac signal (Gained peaks, red) and 2714 peaks with reduced H3K27Ac levels (Lost peaks, blue) in *Arid1a cKO* compared to control hearts (Wald test with Benjamini-Hochberg adjusted *P*-values (false discovery rate (FDR)) < 0.05). **B** Representative example of H3K27Ac signal within the *Sytl2* genomic locus in two replicates (rep) of P7 control (blue) and *Arid1a cKO* (red) hearts. Peaks are shown below (not changed: gray; peaks with increased H3K27Ac in *Arid1a cKO* hearts: red). **C** Gene ontology analysis for biological processes associated with lost peaks. Cardiac muscle development and contraction, and ion handling are enriched. **D** Average H3K27Ac peak signals (top) and heatmaps for genes overexpressed (middle) or underexpressed (bottom) in *Arid1a cKO* hearts compared to controls. H3K27Ac in overexpressed genes is increased in *Arid1a cKO* hearts, whereas H3K27Ac in underexpressed genes is decreased compared to controls. **E** Intersection of RNA-Seq and H3K27Ac ChIP-Seq indicates that lost peaks are associated with downregulated genes, whereas gained peaks are linked to overexpressed genes. **F** Motif enrichment analysis shows transcription factor motifs enriched in lost peaks (left, blue) and gained peaks (right, red), ranked by increasing *P*-value (Hypergeometric Optimization of Motif EnRichment (HOMER) determined *P*-values of motif enrichment). **G** Distinct DNA binding motifs of enriched factors in gained peaks and lost peaks. Source data are provided as a Source Data file.

The SWI/SNF chromatin remodeling complex has critical functions in the establishment and maintenance of the cardiac epigenetic landscape^20,29^. Furthermore, recent studies have proposed ARID1A as a regulator of chromatin accessibility through histone H3 lysine 27 acetylation (H3K27Ac)^39–41^. To investigate the roles of ARID1A in the neonatal cardiac epigenome, we performed ChIP-Seq for H3K27Ac and compared *Arid1a cKO* mutant and littermate control hearts (Supplemental Fig. 4A–D). Differential binding analysis identified 2714 regions with decreased H3K27Ac levels in *Arid1a cKO* mutants (FDR < 0.05; Fig. 5A, B), while 282 had gained H3K27Ac levels (red regions in the ‘peaks’ track in Fig. 5B).

Functional annotation of differential H3K27Ac peaks revealed that lost peaks were preferentially associated with cardiac muscle tissue development, regulation of ion transport, and regulation of heart contraction (Fig. 5C; Supplementary Data 1). Although gained peaks were too few to yield enriched biological processes, there was some enrichment for genes with growth factor activity (GO:MF) and extracellular matrix proteins (GO:CC; Supplementary Data 1). Next, we intersected our RNA-Seq and H3K27Ac ChIP-Seq datasets. When assessing H3K27Ac status near genes that were differentially expressed between *Arid1a cKO* and control hearts, we noticed that underexpressed genes had relatively lower H3K27Ac levels in mutants, whereas overexpressed genes had increased H3K27Ac levels (Fig. 5D). Similarly, peaks with decreased H3K27Ac were preferentially associated with underexpressed genes (10.0%) compared to overexpressed genes (1.4%), and peaks with gained H3K27Ac were often associated with overexpressed genes (20.5%; Fig. 5E). Collectively, these results revealed a clear correlation between H3K27Ac levels and gene expression changes in *Arid1a cKO* hearts versus controls, and provide further support for the hypothesis that *Arid1a* regulates postnatal maturation in cardiac myocytes at the epigenetic level.

### ARID1A acts through distinct transcriptional regulators

To identify the transcriptional regulators involved in driving differential gene expression in *Arid1a cKO* hearts, we performed unbiased motif discovery within differentially acetylated regions. This analysis revealed that the top 5 enriched motifs in *Arid1a cKO* H3K27Ac gained regions were transcription factors of the TEA domain family (TEAD) (Fig. 5F, G; Supplementary Data 2). TEAD transcription factors are required cofactors for YAP and WW domain-containing transcription regulator 1 (WWTR1; TAZ), the downstream effectors of the Hippo pathway. We also identified motifs for homeobox proteins (HOXD10, GSC, OTX2), and the transcriptional activator MYCN, an essential regulator of cardiomyocyte proliferation^42^.

In genomic loci with reduced H3K27Ac levels, we detected enriched motifs for MEF2 transcription factors (MEF2C, MEF2D, MEF2B, MEF2A), Estrogen related receptor transcription factors (ERRG, ESRRB), as well as transcription factors involved in epithelial to mesenchymal transition (SLUG, ZEB1, ZEB2). MEF2 transcription factors have multiple roles during all stages of heart development. MEF2A is the predominant MEF2 family member expressed in the postnatal heart, and it activates markers of terminal differentiation while repressing cell cycle genes^43^. Estrogen-related receptors are critical regulators of postnatal cardiac myocyte maturation, activating adult cardiac metabolic and structural genes^44^. Hence, reduced expression of cardiomyocyte maturation genes in *Arid1a cKO* hearts may be regulated by MEF2 and ESRR transcription factors. Notably, qPCR analysis revealed similar expression of MEF2 and ESRR transcription factors between *Arid1a cKO* and control hearts (Supplemental Fig. 4E). Next, we identified predicted MEF2 and ESRR target genes that were suppressed in *Arid1a cKO* hearts (Supplementary Data 3). Amongst these were many key genes driving cardiomyocyte maturation, including the MEF2 target gene *Atp1a2*;^45^ fetal to adult contractile protein switching ESRR-targets *Tnni3* and *Myl2*;^44^ genes driving decreased resting membrane potential of mature cardiomyocytes *Kcnj2* and *Kcnj12*;^46^ and cardiomyocyte maturation-associated RNA splicing factor *Rbfox1*^47^.

Similarly, genes potentially directly activated by enhanced YAP/TEAD activity in *Arid1a cKO* hearts included *Fgf7*, a cardiomyocyte-derived ligand driving myocardial proliferation during development;^48^ *Ankrd1*, a YAP/TEAD target in postnatal cardiomyocytes, overexpression of which is associated with impaired response to increased haemodynamic load;^49,50^ and *Acta2*, a contractile protein that is normally downregulated during postnatal cardiac maturation^51^.

Collectively, our analyses of histone acetylation, gene expression, and motif enrichment indicate that *Arid1a* suppresses YAP/TEAD-regulated cell cycle activity in neonatal cardiomyocytes while stimulating cardiomyocyte maturation via MEF2- and ESRR-dependent pathways.

### *Arid1a* suppresses YAP target gene expression

**Fig. 6:**
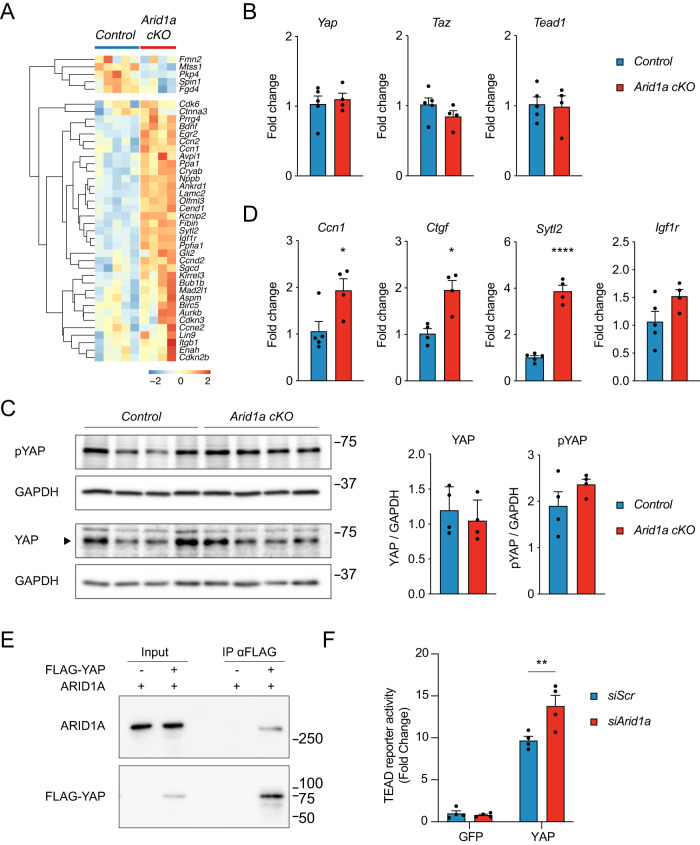
ARID1A directly binds YAP/TAZ and suppresses its activity. **A** Heatmap showing relative expression (z-score) of known YAP-TEAD cardiac target genes in P7 *Arid1a cKO* and control hearts. **B** Relative expression (Fold change vs Control) of *YAP* (*P* = 0.6700), *Taz* (*P* = 0.2239), and *Tead1* (*P* = 0.8537) in P7 *Arid1a cKO* (*n* = 4) and control hearts (*n* = 5) as determined by qPCR (two-tailed Student’s *t*-test). **C** Western blot analysis of total YAP and phosphorylated YAP (pYAP) in P7 control and *Arid1a cKO* hearts. Quantification indicates no difference in YAP (*P* = 0.6700) or pYAP (*P* = 0.2038) expression between control and *Arid1a cKO* hearts (*n* = 4; two-tailed Student’s *t*-test). Molecular weight marker (kDa) is shown at the right. **D** Relative expression of known YAP target genes *Ccn1* (*P* = 0.0295), *Ctgf* (*P* = 0.0111), *Sytl2* (*P* < 0.0001), and *Igf1r* (*P* = 0.0876) in *Arid1a cKO* (*n* = 4) and control hearts (*n* = 4–5) as determined by qPCR (*P < 0.05; ***P < 0.001, ****P < 0.0001, two-tailed Student’s *t*-test). **E** Representative example of 3 independent immunoprecipitations (IP) of FLAG-YAP and ARID1A in H10 cells, showing specific co-precipitation of ARID1A with YAP. Molecular weight marker (kDa) is shown at the right. **F** Luciferase assay with 8xGTIIC-luciferase YAP-TEAD reporter construct in H10 cells (Fold change versus siScr in GFP condition). Co-transfection of siRNA against *Arid1a* significantly induces reporter expression in the presence of YAP (*P* = 0.0028). pCDNA-GFP was used as a control vector. (**, *P* < 0.01. two-way ANOVA with Sidak’s multiple comparison test; *n* = 4 independent experiments). Bar graphs show mean with standard error of the mean. Source data are provided as a Source Data file.

Since *Arid1a cKO* hearts display striking similarities to hearts with forced YAP activation^14,15^, we decided to further explore the molecular mechanism by which ARID1A interacts with Hippo pathway effectors during heart maturation. First, we assessed the expression of known YAP target genes in cardiomyocytes^12,50^ in *Arid1a cKO* hearts and controls. Our RNA-Seq analysis revealed that the majority of known YAP target genes in postnatal hearts were induced in *Arid1a cKO* hearts compared to controls (Fig. 6A). This was not due to differential expression of *Yap*, *Taz*, and *Tead1* in the absence of *Arid1a* (Fig. 6B). Furthermore, quantification of total YAP levels revealed that loss of *Arid1a* did not affect YAP protein levels in P7 hearts (Fig. 6C). YAP can be phosphorylated by the Hippo pathway kinases LATS1 and LATS2, resulting in its cytoplasmic retention and degradation^52^. Therefore, we examined if decreased YAP phosphorylation could account for increased YAP target gene expression. However, also the levels of phosphorylated YAP were similar between *Arid1a cKO* hearts and controls (Fig. 6C). Immunofluorescence staining suggested more pronounced nuclear localization of YAP in mutant hearts (Supplemental Fig. 5A).

Next, we could confirm significant upregulation of YAP target genes, including cellular communication network factor 1 (*Ccn1*), connective tissue growth factor (*Ctgf*), the Rab effector synaptotagmin like 2 (*Sytl2*), and a slight increase of insulin-like growth factor I receptor (*Igf1r*) that did not reach statistical significance (P = 0.0876) by qPCR (Fig. 6D). Hence, these results suggest functional interplay between ARID1A and YAP in neonatal cardiomyocytes.

To further explore the mechanism by which ARID1A interacts with YAP and TAZ during postnatal heart maturation, we evaluated if ARID1A could bind YAP and TAZ in a cardiac-like context. To this end, we overexpressed YAP and ARID1A in heart-derived H10 cells^53^ and performed co-immunoprecipitation and found that ARID1A was efficiently co-precipitated with YAP and TAZ (Fig. 6E, Supplemental Fig. 5A). Next, we assessed whether ARID1A affected YAP activity in cardiac cells using an 8xGTIIc-Luc TEAD reporter construct^54^ and observed potent activation by YAP. Moreover, siRNA-mediated suppression of *Arid1a* significantly enhanced YAP-mediated activation of the reporter, indicating that ARID1A restrains YAP transcriptional activity (Fig. 6F; Supplemental Fig. 5A). Collectively, our analyses indicate that ARID1A modulates YAP transcriptional activity during cardiac maturation, and this may be regulated by direct interaction between ARID1A and YAP.

### ARID1A limits proliferation of border zone cardiomyocytes

Based on our findings that *Arid1a* suppresses cardiomyocyte proliferation in neonatal cardiomyocytes, we next hypothesized that *Arid1a* upregulation in border zone cardiomyocytes after ischemic injury (Fig. 1) may also impede their proliferative capacity. To address this question, we used the tamoxifen-inducible *αMHC-mER-Cre-mER* to ablate *Arid1a* from adult cardiomyocytes (*Arid1a icKO*; Supplemental Fig. 6A–D). We assessed HW and cardiac function by echocardiography and found no signs of remodeling or functional decline, indicating *Arid1a* is not required in cardiomyocytes for normal heart function under baseline conditions (Supplemental Fig. 6E, F).

**Fig. 7:**
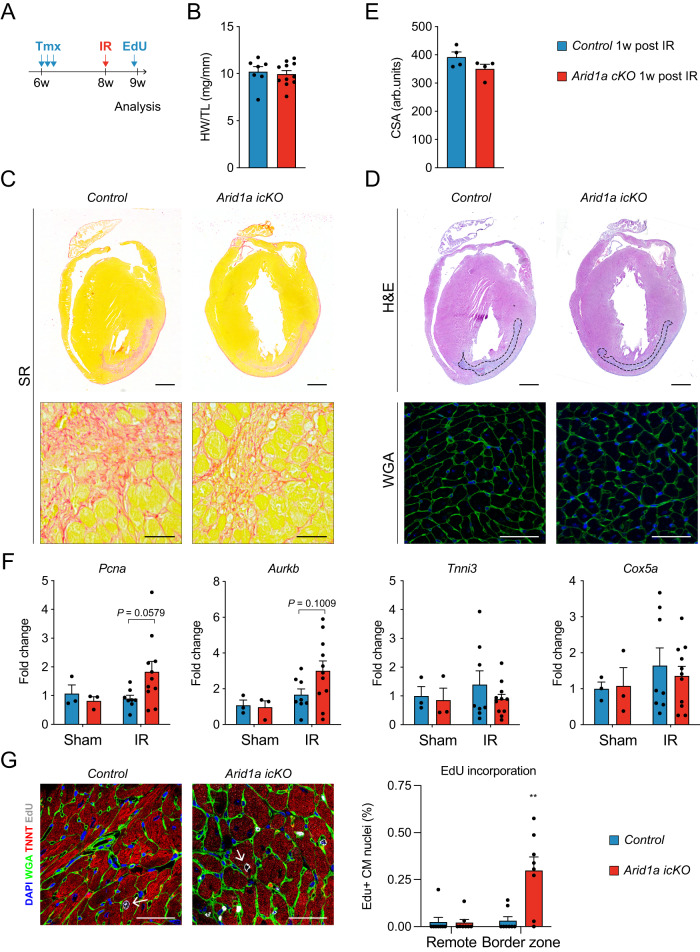
Loss of ARID1A induces cardiomyocyte proliferation after ischemic injury. **A** Schematic showing ischemia-reperfusion (IR) injury timeline. Two weeks after tamoxifen (TMX) administration, IR injury was induced. EdU was injected IP 6 days after IR, and hearts were harvested for analysis 24 h later. **B** *Arid1a icKO* hearts (*n* = 11) have similar heart weight to tibia length (HW/TL) to controls (*n* = 7) 1 week (w) after IR (*P* = 0.7149; two-tailed Student’s *t*-test). **C** Representative examples of Sirius red (SR) staining for fibrosis (red, *n* = 4). **D** Hematoxylin and eosin (H&E) staining for gross tissue morphology and wheat germ agglutinin (WGA) staining for assessment of cardiomyocyte cross sections area (CSA), as quantified in (**E**) reveals no differences between *Arid1a icKO* and control hearts 1w after IR (*n* = 4; *P* = 0.4857; two-tailed Student’s *t*-test). **F** qPCR of key cell cycle, contraction and metabolism genes in *Arid1a icKO* and control hearts subjected to Sham (*n* = 3) or IR surgery (Control, *n* = 8; *Arid1a icKO*, *n* = 11). Expression in infarct regions for cell cycle regulators *Pcna* (*P* = 0.0579) and *Aurkb* (*P* = 0.1009) trends towards being increased in in *Arid1a icKO* compared to controls, *Tnni3* (*P* = 0.4331) and *Cox5a* (0.8074) do not (two-way ANOVA with Šídák’s multiple comparisons test). **G** Representative example of EdU incorporation in control and *Arid1a icKO* IR hearts and quantification of EdU incorporation. EdU incorporation is enhanced specifically in *Arid1a icKO* border zone cardiomyocytes (*P* = 0.0044; *n* = 8; **P < 0.01; Conway-Maxwell Poisson regression analysis). Scalebars 1 mm (top panels in **C**, **D**), 50 µm (bottom panels in **C**, **D**; **G**). Bar graphs show mean with standard error of the mean. Source data are provided as a Source Data file.

To assess the roles of *Arid1a* in cardiomyocyte proliferation after ischemic injury, we subjected control and *Arid1a icKO* mouse hearts to IR injury (Fig. 7A). Gross cardiac morphology, HW, and cardiomyocyte size (CAS) were not different between *Arid1a icKO* and control hearts (Fig. 7B–E). Using qPCR, we analyzed expression of cell cycle genes that were changed in neonatal cardiomyocytes, including *Pcna*, *Aurkb*, *Ccnb2*, and *Ki67*. Although several genes showed a trend towards being induced in *Arid1a icKO* infarct areas (Fig. 7F and Supplemental Fig. 6G), none of them were significantly changed (two-way ANOVA with Šídák’s multiple comparisons test). Genes involved in contraction and metabolism, including *Tnni3* and *Cox5a*, were not changed (Fig. 7F and Supplemental Fig. 6G).

As cardiomyocyte gene expression changes might be masked by the influx of non-cardiomyocytes into the infarct area, we next used EdU staining to quantify cardiomyocyte proliferation. Hearts were injected with EdU 6 days after injury, and EdU incorporation was quantified in 8 control and 8 *Arid1a icKO* hearts on day 7. This analysis revealed that in *Arid1a icKO* mice subjected to IR, there was a significant increase in EdU incorporation in cardiomyocytes near the infarct zone, which was not observed in remote areas (Fig. 7G, Supplemental Fig. 7). Thus, *Arid1a* suppresses adult cardiomyocyte proliferation after ischemic injury, thereby limiting regenerative potential of border zone cardiomyocytes.

**Fig. 8:**
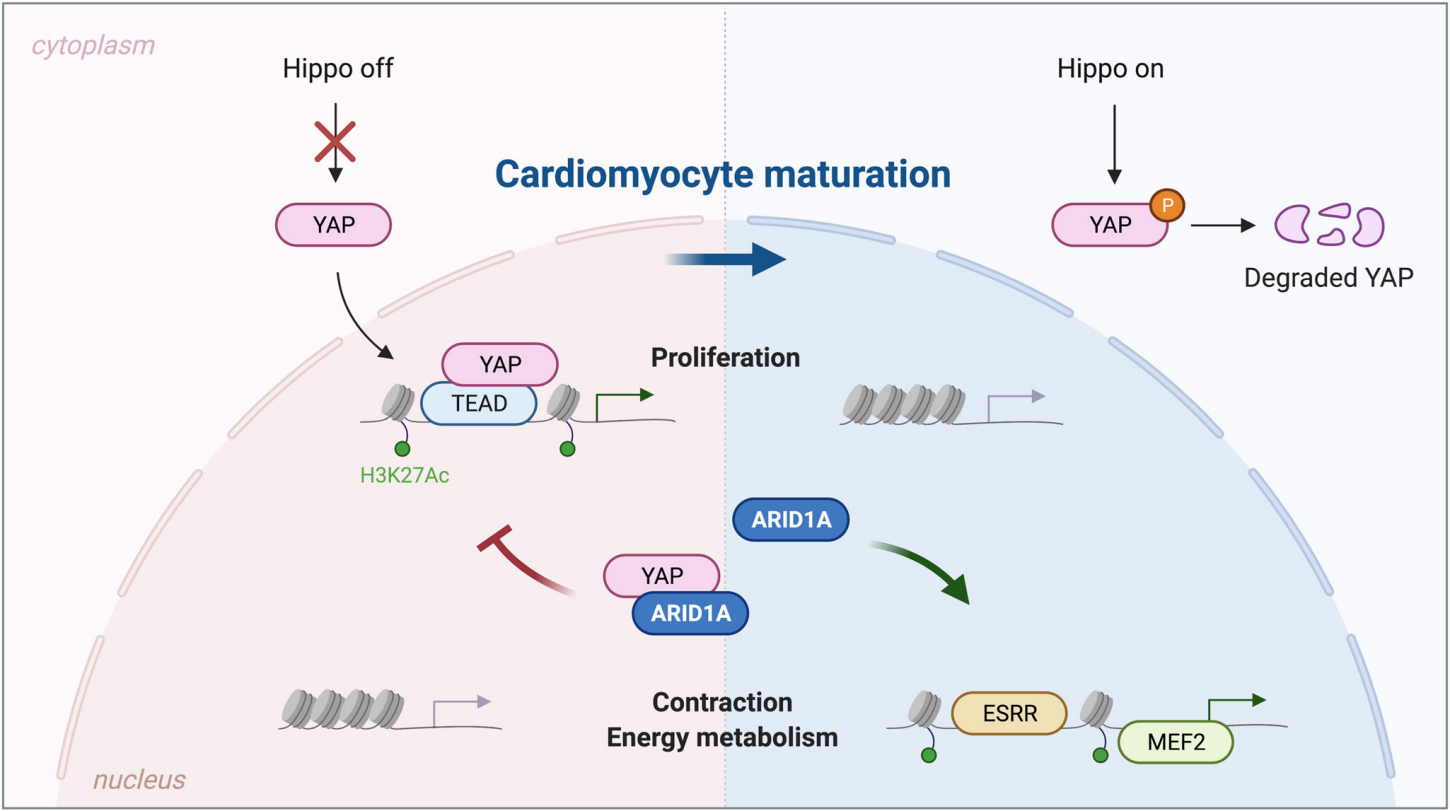
ARID1A regulates cardiomyocyte proliferation through interaction with YAP. In neonatal cardiomyocytes (left), the Hippo pathway is inactive, and YAP translocates to the nucleus where it teams up with TEAD factors to drive a pro-proliferative gene program. Proliferation genes are marked by open chromatin and H3K27Ac histone marks. As cardiomyocytes switch towards maturation, ARID1A binds YAP, inhibiting its transcriptional activity. Proliferation gene program is stably silenced by decreased H3K27Ac levels and transcriptionally inactive heterochromatin. During cardiomyocyte maturation, ARID1A is required for inducing activation of cardiac contractility and metabolism genes, driven by MEF2 and ESRR transcription factors. Created with BioRender.com.

In conclusion, we show that *Arid1a* promotes postnatal cardiomyocyte maturation and suppresses proliferation by interacting with YAP and suppressing its activity (Fig. 8). Even though modulation of *Arid1a* levels is not potent enough to elicit substantial cardiac repair after ischemic injury, *Arid1a* presents itself as a critical suppressor of mouse and human cardiomyocyte proliferation.

## Discussion

The regulation of cardiomyocyte proliferation and maturation are key areas of investigation for cardiac regenerative medicine. Here, we identified *Arid1a* as an essential regulator that selectively drives cardiomyocyte maturation and suppresses proliferation after birth and in response to ischemic injury in vivo. We show that ARID1A antagonizes the activity of nuclear YAP, limiting its effect on cardiomyocyte proliferation. Loss of *Arid1a* leads to loss of active chromatin marks associated with transcription factors driving cardiomyocyte maturation. Hence, our results support a model in which *Arid1a* promotes DNA access of transcription factors that support terminal differentiation and antagonize cell proliferation. The absence of *Arid1a* leaves cardiomyocytes immature with sustained YAP activity and more susceptible to proliferation.

### SWI/SNF functions in heart

ARID1A is a subunit of the SWI/SNF chromatin remodeling complex. SWI/SNF complexes are made up of multiple subunits that are assembled in a combinatorial manner to tailor their functions, regulating specific (cardiac) developmental events^55,56^. These complexes fulfill many essential roles during early specification of mesoderm, formation of cardiac progenitors, and differentiation of embryonic cardiomyocytes^20,24,29,31,55,57,58^. Essential roles during early cardiac development often form a barrier for studying the roles of SWI/SNF subunits during later stages. Here, we used *aMHC-Cre;Arid1a* transgenic mice to induce perinatal ablation of *Arid1a* from cardiomyocytes, thereby overcoming embryonic lethality due to developmental functions. Similar approaches have exposed functions of other SWI/SNF complex subunits in postnatal hearts. The SWI/SNF central ATP-ase brahma related gene 1 (*Brg1*) facilitates myosin heavy chain isoform switching that occurs during cardiac maturation^25^. Furthermore, the cardiac enriched SWI/SNF subunit *Baf60c* regulates a gene expression program that includes genes encoding contractile proteins, modulators of sarcomere function, and cardiac metabolic genes^59^. These functions are in line with the maturation-promoting functions of *Arid1a* we uncovered in the present study, indicating *Arid1a* may act in concert with *Baf60c* during cardiomyocyte maturation. In contrast, cardiomyocyte specific *Baf60c* knockout mice show reduced proliferation, indicating that *Arid1a* regulates cardiomyocyte proliferation via mechanisms independent of *Baf60c*^59^.

### ARID1A modulates the proliferation and maturation balance

This study revealed that *Arid1a* acts as an essential regulator of the balance between proliferation and maturation in postnatal mouse cardiomyocytes, and human iPS-CM. This is in line with its function during embryonic cardiomyocyte differentiation, as well as in other organs such as the liver, where *Arid1a* promotes cell differentiation at the expense of cell renewal^28,29^. Even more, suppression of *Arid1a* in liver and outer ear injury models significantly enhanced tissue regeneration via enhanced proliferation of parenchymal cells^28^. In muscle stem cells, SWI/SNF also functions as a context-dependent regulator of this equilibrium, providing an essential brake to cell cycle progression^60^. In line with its roles in coordinating cell proliferation and differentiation, mutations in SWI/SNF components contribute to an estimated 20% of all cancers, with *Arid1a* being the most frequently mutated subunit^61,62^.

### ARID1A interacts with YAP

Phenotypic analysis of postnatal *Arid1a cKO* hearts combined with RNA-Seq and ChIP-Seq revealed a key role for YAP-dependent signaling. *Arid1a cKO* hearts are overgrown with sustained proliferation, showing striking similarities to postnatal hearts with ectopic YAP activation via knockout of salvador1 (*Sav1*) or through overexpression of constitutively active YAP^10,12^. We observed induction of YAP dependent gene programs in *Arid1a cKO* hearts, while YAP expression level and phosphorylation status, as assessed by Western Blot, were not changed. Still, YAP appeared more nuclear localized in mutant hearts, which is compatible with enhanced expression of YAP target genes. These findings indicate that ARID1A may suppress YAP through a non-canonical mechanism distinct from the canonical regulation of YAP by the Hippo pathway. Recently, in a screen for nuclear factors that interact with YAP, Chang et al. identified the ARID1A-containing SWI/SNF complex as an inhibitor of YAP and TAZ^63^. In a series of elegant in vitro and in vivo experiments, they show that ARID1A competes for YAP-binding with TEAD, providing essential tumor-suppressive function of the SWI/SNF complex. Here, we show that ARID1A can bind YAP and inhibit its transcriptional activity in cardiac like cells, using affinity tagged proteins. Immunoprecipitation of endogenous complexes from neonatal hearts would further strengthen this observation.

On the other hand, ARID1A could mediate chromatin accessibility, providing a chromatin status that allows for the expression of cardiomyocyte maturation gene programs, while silencing regions associated with proliferation. In liver cells, ARID1A-mediated chromatin opening facilitates a chromatin environment that is permissive to response to injury^64^. Upon liver injury, YAP-dependent gene expression is activated, suggesting that ARID1A-opened chromatin renders hepatocytes competent to respond to YAP signaling. Increased H3K27Ac levels of YAP regulated promoters and enhancers in *Arid1a cKO* hearts could suggest that Arid1a restricts chromatin access of YAP dependent genes during cardiac maturation. Taken together, these results suggest that ARID1A may have multiple ways to control YAP-dependent gene expression.

### ARID1A promotes cardiomyocyte maturation

Here, we show that ARID1A is essential for the expression of a maturation-promoting gene program in neonatal mouse heart, as well human iPS-CMs. Moreover, we show that overexpression of ARID1A can enhance features of cardiomyocyte maturation in human EHMs. On the other hand, *Arid1a* appeared dispensable for adult heart function under homeostatic conditions. Moreover, we did not observe deregulation of contraction or metabolism genes in infarct tissue after IR, although changes in cardiomyocyte gene expression could be masked by other cell types.

During postnatal maturation, cardiomyocytes switch their energy metabolism from glycolysis towards fatty acid oxidation. While gene expression changes in *Arid1a cKO* hearts indicate roles of *Arid1a* in this switch, further studies are required to fully understand roles of *Arid1a* in the energy metabolism switch during cardiac maturation. Together, these data indicate *Arid1a* plays important roles during the process of maturation.

Once cardiomyocytes have matured, they may no longer require *Arid1a* to maintain the expression of these gene programs. Our analysis of H3K27Ac in neonatal hearts indicates that ARID1A is required for the establishment of a maturation-promoting epigenetic landscape in cardiomyocytes. In line with its role during cardiomyocyte development, ARID1A may steer cardiac maturation through chromatin remodeling activity, promoting DNA access of pro-maturation factors MEF2A and ESRRB^29,35^. Alternatively, ARID1A could be recruited by MEF2 and ESRR factors, and activate or maintain pro-maturation gene expression through regulating histone H3K27 acetylation levels^39–41^.

### *Arid1a* in cardiac regenerative medicine

Regulation of cardiomyocyte proliferation and maturation could ultimately be exploited for cardiac regenerative applications. For instance, suppression of *Arid1a* might contribute to enhanced tissue regeneration after injury. However, due to its critical role as tumor suppressor, such an approach would have to be transient and cardiomyocyte specific. In addition, the modest increase in proliferation in *Arid1a* ablated adult cardiomyocytes indicates additional factors are required to fully activate proliferation. Suppression of *Arid1a* in the context of YAP-induced cardiomyocyte proliferation^10,65^ might enhance proliferation rates, whereas induction of *Arid1a* at later stages could be used to limit YAP activity, preventing over-proliferation. Finally, maturation-promoting functions of *Arid1a* could be utilized for in vitro cardiomyocyte maturation and cardiac tissue engineering efforts.

Together, our studies reveal essential roles of *Arid1a* in the regulation of proliferation and maturation, providing further insights into cardiomyocyte biology. Future studies will be required to discover the full potential of modulating *Arid1a* levels for cardiac regenerative applications.

## Methods

### Mouse studies

Animal studies were approved by the animal welfare agency (I.v.D.) of the Royal Dutch Academy of Sciences and Arts (K.N.A.W.) and in compliance with national legislation and institutional guidelines. *Arid1a*^*loxP*^ (*Arid1a*^*tm1.1Zhwa/J*^; Stock#: 027717)^57^, *Rosa26*^*tdTomato*^ (*Rosa26*^*tm14(CAG-tdTomato)Hze*^; Stock#: 007914)^66^ and wildtype C57B/6 J (Stock#: 000664) mice were obtained from Jackson Laboratories. *αMHC-Cre*^36^, and *αMHC-mER-Cre-mER*^67^ transgenes were obtained from M.D. Schneider (Imperial College, London, UK) and J.D. Molkentin (University of Cincinnati, OH, USA), respectively. Mouse lines were maintained on C57B/6 J background. *αMHC-mER-Cre-mER* was induced with Tamoxifen (Cayman Chemical Company, cat# 13258) by daily intraperitoneal injection at 30 mg/kg bodyweight for 3 consecutive days. Male and female mice were included in studies performed in pups, studies in adult mice only included males.

### Ischemic injury

For ischemia-reperfusion (IR) injury, mice were anaesthetized with a mix of Fentanyl (0.05 mg/kg), Midazolam (5 mg/kg), and Dexmedetomidine (0.125 mg/kg) via intraperitoneal injections. Animals were artificially ventilated during surgery while kept on a heating pad at 38 °C. Buprenorphine was injected subcutaneously as analgesic (0.1 mg/kg) before surgery, after 10 h, and after 24 h. IR was induced by a 1 h ligation of the left anterior descending artery (LAD) by placing a 7–0 silk suture around the LAD together with a 2–3 mm PE 10 tubing. Reperfusion of the myocardium was achieved by cutting the ligature. For sham surgery, a similar procedure was performed with the exclusion of a 1 h ligation of the LAD artery. Permanent LAD occlusion, referred to as myocardial infarction, was performed as described previously^68^.

### Transthoracic echocardiography (echo)

Cardiac function was evaluated by two-dimensional transthoracic echocardiography on sedated, adult mice (2% isoflurane) with a MS400-0257 transducer for adults, or MS700-0112 for pups (VisualSonics Inc., Toronto, Canada). Hearts were imaged in parasternal long-axis and short-axis views at the level of the papillary muscles, to record M-mode measurements, determine heart rate, wall thickness, and end-diastolic and end-systolic dimensions. Data was analyzed using Vevo Lab software (version 5.7.1). Fractional shortening (end-diastolic dimension minus end-systolic dimension normalized for end-diastolic dimension) as well as ejection fraction (stroke volume normalized for the end-diastolic volume), were used as an index of cardiac contractile function.

### In vivo EdU labeling

5-ethynyl-2´-deoxyuridine (EdU) was injected subcutaneous (neonates; at P1, P3 and P5) or intraperitoneal (adult; at 1, 3, 5 days after IR or sham surgery) at 50 mg/kg body weight. EdU was detected in histological sections using Click-iT EdU Cell Proliferation Kit (Thermo Fisher Scientific). EdU incorporation in cardiomyocyte nuclei (Tnnt2^+^, DAPI^+^) was quantified using Fiji (version 1.53a)^69^.

### QPCR

Total RNA was extracted from hearts and iPS-CMs using standard TRIzol extraction (Thermo Fisher) and reverse transcribed using iScript cDNA synthesis kit (Bio-Rad, 1708891). Real time quantitative PCR (qPCR) was performed using IQ Sybr-Green supermix (Bio-Rad, #170-8884), CFX Connect real time PCR detection system (Bio-Rad). Gene expression was quantified using ddCT method with *Gapdh* (mouse) or *ARP* (human) as housekeeping gene and a minimum of 3 samples per condition as detailed in figure legends.

### Western blot

Heart tissue was snap frozen and stored at −80C, then thawed and homogenized using a micropestle, iPS-CMs were collected by cell-scraping in RIPA buffer supplemented with Protease inhibitor cocktail (Roche, #11836170001). Samples were denatured in 4x Leammli buffer, supplemented with 2% β-mercaptoethanol for 5 min at 99 °C and subject to SDS-PAGE and western blotting using standard procedures. In case not all samples from one experiment fit one gel, samples of different conditions were distributed equally over multiple gels. Antibodies used are listed in Supplemental Table 3. Blots were visualized using Clarity Western ECL Substrate (Bio-Rad, #170-5061) and Amersham Imager 600. Stripping was performed using mild stripping buffer (1.5% glycine, 0.1% SDS, 1% Tween, pH 2,2). GAPDH (38 kDa), or Vinculin (123 kDa) were used as loading control.

### Histology and immunostaining

Hearts were flushed with PBS and fixed with 4% Formalin for 48 h at RT and embedded in paraffin. 4 μm sections were subjected to standard Hematoxylin and Eosin (H&E) or Sirius Red (SR) staining. For immunostaining, sections were subjected to antigen retrieval by boiling 20 min in 10 mM Tris, 1 mM EDTA, pH 9 and slowly cooling down to RT. Sections were blocked with 0,05% bovine serum albumin (BSA) in PBST (PBS with 0,1% Tween-20) for 1 h at RT. Primary antibody incubations were performed o/n at 4 °C, secondary antibody incubations were performed for at least 2 h at RT, using the antibodies and dilutions as listed in Supplemental Table 2. 4′,6-diamidino-2-phenylindole (DAPI, 5μM) was used to stain nuclei. Wheat germ agglutinin (WGA; Merck, Cat# FITC conjugate 100 ug/mL) was added during secondary antibody incubation for the staining of cardiac sarcolemma to determine cross sectional area.

Images were acquired on a Leica SPE or SP8 confocal microscope using LAS X software (Leica, version 3.3.0 or newer), or Olympus Slideview VS200. Objectives used for imaging are listed in Supplemental Table 3. Linear adjustment of contrast, brightness or color was applied using Fiji (version 1.35a), and was applied to entire images and equally to all conditions within the same experiment.

### WGA quantification

Cardiomyocyte cross-sectional area (CSA) was determined using WGA counterstaining in DAPI+ cardiomyocytes by manual tracing of cross-sectioned, nucleated, cardiomyocytes using the ‘measure’ function in Fiji (version 1.53a)^69^.

### RNA-seq

Total RNA was extracted from 4 *Arid1a*^*loxP/loxP*^ controls and 4 *αMHC-Cre;Arid1a*^*loxP/loxP*^ mutants using standard TRIzol extraction (Thermo Fisher). Illumina TruSeq stranded mRNA libraries were sequenced single end 75 base pairs on Illumina NextSeq500. Reads were mapped to mouse genome (mm10) and differential expression analysis was performed using DeSeq2 1.24.0^70^. Differentially expressed genes were those with absolute fold change of 1.5 and adjusted Benjamini-Hochberg adjusted *P* < 0.05. Functional annotation was performed using fGSEA 1.5.2 with molecular signatures database (MsigDB; version 6.2)^71^.

### ChIP-seq

Chromatin isolation and H3K27Ac ChIP-Seq was performed on 2 *Arid1a*^*loxP/loxP*^ controls and 2 *αMHC-Cre;Arid1a*^*loxP/loxP*^ mutant P7 ventricles as described previously^72^. Briefly, P7 hearts were minced on ice using scalpels, and taken up in 5 ml 1.5% paraformaldehyde at room temperature (RT). Hearts were homogenized after 5 min using ultraturrax T18 homogenizer (IKA) at setting 4 for 30 sec and incubated for 5 more min at RT. Crosslinking was quenched using Glycine (125 mM final concentration). Nuclei were extracted and chromatin was sheared using an S2 focused ultra-sonicator (Covaris) at 5% duty cycle, intensity 3 and 200 cycles per burst at 4 °C. Immunoprecipitation was performed overnight at 4 °C using anti-H3K27Ac (Active Motif, Cat# 39133, 5 μg/sample) coated Dynabeads ProtG (Thermo Fisher, Cat# 10004D, 50 μl/sample).

Libraries were generated for input and IP of 2 *Arid1a*^*loxP/loxP*^ controls and 2 *αMHC-Cre;Arid1a*^*loxP/loxP*^ mutant hearts. Reads were mapped to mouse genome (mm10) using Burrows-Wheeler Aligner (BWA, version 0.7.17-r1188) and peaks were called using Homer findPeaks (v4.10)^73^ with setting -style histone, using merged input reads from all samples as input. H3K27Ac signals were detected at the genomic loci of cardiac marker genes, such as *Myh6*, *Tnnt2*, *Tbx20*, and *Gata4*, at similar levels in replicate samples, validating data reproducibility (Supplemental Fig. 4A). We identified 45987 H3K27Ac regions (peaks), excluding peaks that were only detected in a single sample. The majority of peaks were located in introns (50%) and intergenic regions (31%), with only 11% mapping to promoters (Supplemental Fig. 4B–D). Differential peaks were called using Diffbind 2.12.0^74^ and Deseq2 1.24.0^70^, using peaks that were present in at least 2 samples, with the false discovery rate threshold set at 0.05. Differential peaks were annotated with StringDB v11.0 (http://ww.stringDB.org) gene ontology biological process (GO:BP), using all H3K27Ac regions as background. Heatmaps for Fig. 4D were generated using the computeMatrix (3.3.0.0.0) and plotHeatmap (3.3.0.0.1) functions of the Deeptools package (version 3.3.0)^75^ on usegalaxy.org^76^. Motif discovery was performed using Homer findMotifsGenome package (version 4.11) on gained or lost H3K27Ac regions using all H3K27Ac regions as background.

### Co-immunoprecipitation

Rat heart derived H10 cells^53^ seeded in 10 cm dishes were transfected with 10μg ARID1A expression construct plus 5μg FLAG-YAP, 5μg FLAG-TAZ, or 5μg pcDNA6 empty vector control. 48 h after transfection, cells were lysed on ice in lysis buffer (50 mM HEPES (pH 7.5), 100 mM NaCl, 50 mM KCl, 1% Triton X-100, 5% glycerol, 0.5% NP-40, 2 mM MgCl2, and 1 protease inhibitor tablet) and cleared by centrifugation at 16,000 g for 15 min. Extracts were incubated overnight with anti-FLAG beads (Sigma-Aldrich, Cat# M8823) with gentle mixing at 4 °C. Beads were washed three times with lysis buffer on ice, and eluted using 3xFLAG peptide (Sigma-Aldrich, Cat# F4799) 5 μg/μl in 150 mM NaCl, 50 mM Tris-HCl, pH 7.5. Representative examples of at least 3 independent experiments are shown.

### Luciferase assay

H10 cells were seeded in 24-wells plates and transfected with 0.1 nM siArid1a (OriGene, Cat# SR510109) or siScr (OriGene, Cat#SR30004) after 24 h using Lipofectamine RNAiMax (Invitrogen). On day 2, cells were transfected with 100 ng 8xGTIIC-luciferase, 10 ng TK-Renilla, and 6 ng YAP, TAZ or control vector (pcDNA-GFP) per well. On day 4, luciferase activity was measured using Dual-Luciferase® Reporter Assay System (Promega, # E1910) and the Berthold Centro LB 960 luminometer according to manufacturers instructions.

### Plasmid constructs

pcDNA6-ARID1A was a gift from Ie-Ming Shih (Addgene plasmid # 39311). 8xGTIIC-luciferase construct was a gift from Stefano Piccolo (Addgene plasmids # 34615). To minimize the effect of Hippo pathway-induced cytoplasmic localization of YAP, we used the constitutively nuclear 2xFLAG-YAP-5SA, which was generated by reverting the S94A mutation from Addgene plasmid #33101. 2xFLAG-TAZ was generated by cloning the human WWTR1 coding sequence (NM_015472.6) without ATG into the *KpnI* and *XbaI* sites in pCMV-2xFLAG. psPAX2 and pMD2.G were a gift from Didier Trono (Addgene plasmid # 12260 and # 12259, respectively). pLVX-eGFP was obtained by cloning PCR amplified eGFP with extended primers containing XhoI (FW) and NotI (RV) sites into XhoI, NotI digested pLVX-IRES-Hygro (Clontech). pLVX-ARID1A was obtained by cloning ARID1A-V5-HIS6 from pcDNA-ARID1A with NheI (compatible with SpeI) and PmeI (blunt) into the 6.9 kb SpeI and MluI (T4 polymerase blunted) backbone of pLVX-IRES-Hygro.

### Cardiomyocyte differentiation from iPS cells

For directed differentiation towards cardiomyocytes, human induced pluripotent stem cells (hiPSCs; Healthy, White, 31-year-old, male donor; ATCC-BYS0112) were cultured on Geltrex-coated plates in Essential 8 Medium until 80%–90% confluency. Next, cells were cultured in cardio differentiation medium [RPMI-1640-Medium-GlutaMAX Supplement-HEPES (GIBCO, 72400-021) supplemented with 0.5 mg/mL human recombinant albumin (Sigma-Aldrich, A9731), 0.2 mg/mL L-Ascorbic Acid 2-Phosphate (Sigma-Aldrich, A8960)], with 4 μM CHIR99021 (Sigma-Aldrich, 361559).After 48 h, medium was refreshed with cardio differentiation medium with 5 μM IWP2 (Sigma-Aldrich, 681671). After 48 and 96 h, cells were refreshed with plain cardio differentiation medium. From day 8 onward, cells were kept in RPMI-1640-Medium-GlutaMAX-Supplement-HEPES supplemented with B-27 Supplement (50x)-serum free (GIBCO, 17504001) and spontaneous beating could be observed starting from day 8–10. hiPSC-derived cardiomyocyte (iPS-CM) culture purity was assessed by the percentage of cardiac Troponin T positive cardiomyocytes (Abcam, ab45932; 1:2000).

### iPS-CM experiments

HEK293T Lenti-X cells (TaKaRaBio, Cat# 632180) were transfected with pSPAX2, pMD2.G, and pLVX-eGFP or pLVX-ARID1A to generate virus. Medium was harvested after 48 h, and relative virus titers were determined by qPCR. iPS-CMs were transduced with Lenti-GFP or Lenti-ARID1A by adding the virus to the medium, and incubating for 4 days, when RNA and protein were isolated. Human siRNA against ARID1A (siARID1A) and scrambled siRNA (siScr) were obtained from Origene (Cat# SR305421 / SR30004). iPS-CMs were transfected using Lipofectamine RNAiMax (Invitrogen) at day 0 and again at day 2, cells were collected for analysis at day 4. For EdU analysis, iPS-CMs were seeded onto GelTrex coated coverslips, and subsequently treated with siRNA or Lenti-virus as described. EdU was added to the cells at day 2 and incubated for 48 h. Cells were fixed using 2% paraformaldehyde. EdU incorporation was quantified using positive cell detection in QuPath v0.4.0 (nucleus parameters: background radius 8μm, sigma 1.5 μm, minimum area 40 μm^2, maximum area 400 μm^2, threshold 100; positive nucleus parameters: score compartment nucleus Alexa647 mean, threshold 500).

### EHM generation

EHM was generated as described previously^18^. In brief, iPSC-derived CMs and human foreskin fibroblasts (HFF) (HFF-1, ATCC, SCRC-1041) were mixed at a ratio 70:30, resuspended in Collagen type I (Collagen Solutions, FS22024) diluted into RPMI 2x (Thermo Fisher Scientific, 51800-035) and then cast into wells of an EHM multi-well plate (Myriamed GmbH, myrPlate-TM5). After 45 min, EHM medium freshly supplemented with TGFβ1 (Peprotech, AF-100-21C) was added, and refreshed daily for the first 3 days. Subsequently, the tissue medium was replaced daily with EHM medium for the entirety of the experimental duration.

### Contraction analyses

Contraction measurements were performed using video-optic analysis of EHM mediated pole bending in a myrPlate-TM5 culture format at 37 °C^38^. Data from spontaneously contracting EHM was recorded for at least 2 min at 50 fps at the indicated time-points in a myrImager prototype (Myriamed GmbH). Percent pole bending is reported as a surrogate for force of contraction (F); contraction and relaxation times are recorded from 20 to 80% peak contraction and 20 to 80% relaxation; contraction and relaxation velocities are reported as maximal and minimal dF/dt.

### Statistics

The number of biological replicates (*n*) used in each experiment is indicated in the figure legends. For iPS-CMs, *n* represents different wells, the number of independent differentiations is indicated with each experiment. For H10 cells, *n* represents independent experiments, each of which is performed at least in triplicate. Results are presented as the mean ± standard error of the mean (SEM). Statistical analyses were performed using PRISM (Graphpad Software Inc, Version 9.5.1). For normally distributed data, as assessed by Kolmogorov-Smirnoff test, two-tailed Student’s *t*-test was used to analyze comparisons between two groups, Mann–Whitney test was used in case normal distribution could not be confirmed. One-way ANOVA with Dunnett’s test for multiple comparisons was used for comparing multiple groups to a control condition. Two-way ANOVA with Sidak’s multiple comparisons test was used to analyze comparisons with two variables. EdU incorporation after IR was analyzed using mean-parametrized Conway-Maxwell Poisson regression analysis as described previously^77^. EdU labeled cell counts were modeled as a function of location (remote or border zone) and animal genotype (control and *Arid1a icKO*). The total number of DAPI+ cardiomyocytes was used as an offset value in the regression equation. Analyses revealed that location or genotype alone had no significant effect on counts of EdU labeled cardiomyocytes, whereas the interaction between location and genotype had a significant effect. Factor correction was used to correct for variation between independent differentiations in qPCR, Western blot and EdU experiments in iPS-CMs^78^. Unless stated otherwise, *P* < 0.05 was considered significant.

### Reporting summary

Further information on research design is available in the Nature Portfolio Reporting Summary linked to this article.

### Supplementary information

Supplementary Information

Description of Additional Supplementary Files

Supplementary Data 1

Supplementary Data 2

Supplementary Data 3

Reporting Summary

### Source data

Source Data

## Data Availability

RNA-Seq and ChIP-Seq data are deposited to gene expression omnibus (GEO) with accession number GSE155102. All uncropped gels and numerical values are provided in the Source data file. Mouse mm10 reference genome used in this study is available from http://hgdownload.cse.ucsc.edu/goldenpath/mm10/bigZips/mm10.fa.gz. Source data are provided as a Source Data file. Source data are provided with this paper.
