# Supplementary material for: Cardiomyocyte proliferation is suppressed by ARID1A-mediated YAP inhibition during cardiac maturation
Source: Nat Commun. 2023 Aug 5;14:4716. doi: 10.1038/s41467-023-40203-2 (PMC10404286; doi:10.1038/s41467-023-40203-2)
Supplement: Supplementary file 1 — Supplementary Information [file 41467_2023_40203_MOESM1_ESM.pdf]

Supplement

Supplemental Tables

Supplemental Table 1. qPCR primer sequences.

Gene	Species	Forward (5’-3’)	Reverse (5’-3’)
Arid1A	Mouse	TGGAGGACAGATGCACTCG	CATTGGGGTAGTTGGCGTTG
Arid2	Mouse	CATAGAGCAGGTCCAAACCC	CCACTGACAAGCGAACTTCT
Atp1a2	Mouse	CTGGGCCGAAAATACCAAGT	CGACAGAACTTGACCCACTC
Aurkb	Mouse	AAATGGTAGATCTATGGTGCATCG	GAACTTCAGGTCCACCTTGACAAT
SmarcD3	Mouse	GCAAAGCCACGAAAAGCAAA	TGCCCATGTACGGGGAG
SmarcA4	Mouse	GGAGGTAGACTACAGCGACT	AAGATTTCTTCTGCCGGACC
Ccn1	Mouse	GGGTTGGAATGCAATTTCGG	CTGGTGTTTACAGTTGGGCT
Ccnb1	Mouse	TTCATGCAGAACAGTTGTGTGC	TTAGTCACAAAGGCGAAGTCACC
Ccnb2	Mouse	AAATGTCAACAAGCAGCCGA	ATCAGAGAAAGCTTGGCAGAG
Cox5a	Mouse	CAGACATTGATGCCTGGGAA	ATTTTGGGCTCAGGAACCAG
Ctgf	Mouse	CTCCACCCGAGTTACCAATG	GTGTCCGGATGCACTTTTTG
Esrra	Mouse	CTCAGCTCTCTACCCAAACG	GGACAGCTGTACTCGATGC
Esrrg	Mouse	CTGGGTACCACTATGGGGTT	TCTCACATTCATTCGTGGCT
Gapdh	Mouse	TGTCGTGGAGTCTACTGGTG	ACACCCATCACAAACATGG
Igf1r	Mouse	ACGAGTGGAGAAATCTGTGGG	CGATCACCGTGCAGTTTTCC
Ki67	Mouse	CCTTTGCTGTCCCCGAAGA	GGCTTCTCATCTGTTGCTTCCT
Mef2A	Mouse	CGAGCATGCTGTCTCCACCC	ATCCCACTTGCACTGCCGGT
Mef2C	Mouse	AGCGCAGGGAATGGATACGG	CATAGGGGGAGGAGATTTGGCT
Myl2	Mouse	ATGACCTAAGGGACACATTTGC	AGGATCAGCCCCTTTAAGTTTC
Pcna	Mouse	TCGGGTGAATTTGCACGTAT	AACGTTAGGTGAACAGGCTC
Sytl2	Mouse	GGGCAGCCACCTAAATTCTT	CAGGCCTGAACCCATCATAC
Taz	Mouse	GGAACCTGAAGTTGATGCGT	AGTCCATCCCTTTCTGGTAGA
Tead1	Mouse	ACATCAAACTCAGGACGGGA	TCCTTCTGGCAAGAACCTGAA
Tnni3	Mouse	CCAGCCTTTGGAGTTGGATG	AACTTGCCACGGAGGTCATA
Yap1	Mouse	ATTTCGGCAGGAATTAGCTCT	CTGTCTGTGCTCTCATCTCG
Wpre	Lenti	CGCTATGTGGATACGCTGCT	CGGGCCACAACTCCTCATAA
ARID1A	Human	CCGAATCTCATGCCTTCCAA	TTGGGTGGAGAACTGATTGC
ARP	Human	CACCATTGAAATCCTGAGTGATGT	TGACCAGCCCAAAGGAGAAG
ATP2A2	Human	ACCCACATTCGAGTTGGAAG	CCAACGAAGGTCAGATTGGT
COX5A	Human	CATTGATGCTGCTTTGCGGG	AGGTCCTGCTTTGTCCTTAACA
COX6C	Human	TAGTCAGGAAGGACGTTGGT	ATAGCACGAATGCTACAGCC
CPT1B	Human	ATCTACCTTCGAGGCAGGAG	GTCCAGTTTACGGCGATACA
GJA1	Human	TTAAGGATCGGGTTAAGGGAAAG	TGTACCCAGGAGGAGACATAG
MYH7	Human	CACTTGAGTAGCCCAGGCACA	CCGCTCCTTCTCTGACTTGC
MYL2	Human	CCTTTCCACCATGGCACCTA	AAGCCATCCCTGTTCTGGTC

PLN	Human	GTCCAATACCTCACTCGCTCAG	TCACGATGATACAGATCAGCAAG
RYR2	Human	GCTGGTTGTGGACTGCAAAG	AACACTCAGGCCTCCGAATG
TNNI3	Human	CTGCGGAGAGTGAGGATCTC	GATGTTCTTGCGCCAGTCTC

Supplemental Table 2. Antibodies.

**Protein**<br>Provider<br>Cat#	Dilution*	Validation
**ACTN2**<br>Sigma-Aldrich<br>A7732	1:200 (IF)	Supplier: Species reactivity include human and mouse. Suitable for IF
**ARID1A**<br>Abcam<br>AB182560	1:200 (IF)<br>1:1000 (WB)	Data in supplemental figures 2B, 2C, 6B and 6E confirm the sensitivity and specificity of this antibody for mouse in both IF and WB.
**Vinculin**<br>Santa Cruz<br>sc-25336	1:1000 (WB)	Supplier: Species reactivity include human and mouse. Suitable for WB
**FLAG**<br>Sigma-Aldrich<br>F3165	1:1000 (WB)	Supplier: Monoclonal ANTI-FLAG® M2 antibody has been used in: immunoblotting, immunoprecipitation
**GAPDH**<br>Millipore<br>MAB374	1:5000 (WB)	Supplier: Species reactivity include human and mouse. Suitable for WB
**PLN**<br>ThermoFisher<br>MA3-922	1:1000 (WB)	Supplier: Species reactivity include human and mouse. Suitable for WB
**tdTomato**<br>SciGen<br>AB8181	1:500 (IF)	Supplier: Suitable for immunohistochemistry - paraffin sections.
**TNNI**<br>Abcam<br>AB47003	1:1000 (WB)	Supplier: Species reactivity include human and mouse. Suitable for WB
**TNNT2**<br>Abcam<br>AB8295	1:200 (IF)	Supplier: Species reactivity include human and mouse. Suitable for IF
**YAP1**<br>Cell Signaling<br>4912S	1:1000 (WB)	Supplier: Species reactivity include human and mouse. Suitable for WB
**YAP1**<br>Novus<br>NB110-58358	1:200 (IF)	Supplier: Species reactivity include human and mouse. Suitable for IF
**YAP1 p-SER127**<br>Cell Signaling<br>13008P	1:1000 (WB)	Supplier: Species reactivity include human and mouse. Suitable for WB
**H3K27Ac**<br>Active Motif<br>39133	1:50 (ChIP)	Supplier: Species reactivity includes mouse. ChIP-grade antibody.

* IF: Immunofluorescence, WB: Western blot, ChIP: Chromatin Immunoprecipitation

Supplemental Table 3. Objectives used for imaging.

Figure panel	Description of the image	Objective	Magn.	NA	IM
1E-left	ARID1A expression E12.5	ACS APO 63.0x/1.30 OIL	63.0x	1.30	Oil
1E-middle	ARID1A expression P7	ACS APO 63.0x/1.30 OIL	63.0x	1.30	Oil
1E-right	ARID1A expression adult	ACS APO 63x/1.30 OIL	63.0x	1.30	Oil
1G-left	3d IR	HC PL FLUOTAR 20.0x/0.50 DRY	20.0x	0.50	Dry
1G-right	3d IR zooms	ACS APO 63.0x/1.30 OIL	63.0x	1.30	Oil
2B	P7,P14 HE overview	HC PL FLUOTAR 10x/0.30 DRY	10.0x	0.30	Dry
2C	P7,P14 HE zooms	HCX PL FLUOTAR 40x/0.75 DRY	40.0x	0.75	Dry
2E-top	Sirius red	HCX PL FLUOTAR 40x/0.75 DRY	40.0x	0.75	Dry
2E-bottom	WGA	ACS APO 40.0x/1.15 OIL	40.0x	1.15	Oil
3E	P7 EdU	ACS APO 63x/1.30 OIL	63.0x	1.30	Oil
4C	EHM brightfield and fluorescent images	Leica plan APO 1.0x	1.0x	--	n/a
7C-top	Adult IR Sirius red overview	HC PL FLUOTAR 10x/0.30 DRY	10.0x	0.30	Dry
7C-bottom	Adult IR Sirius red zooms	HCX PL FLUOTAR 40x/0.75 DRY	40.0x	0.75	Dry
7D-top	Adult IR HE overview	HC PL FLUOTAR 10x/0.30 DRY	10.0x	0.30	Dry
7D-bottom	Adult IR WGA zooms	HC PL APO CS2 63x/1.40 OIL	63x	1.40	Oil
7G	Adult EdU zooms	HC PL APO CS2 63x/1.40 OIL	63x	1.40	Oil
S1A-left	ARID1A expression E12.5	ACS APO 63.0x/1.30 OIL	63.0x	1.30	Oil
S1A-middle	ARID1A expression P7	ACS APO 63.0x/1.30 OIL	63.0x	1.30	Oil
S1A-right	ARID1A expression adult	ACS APO 63x/1.30 OIL	63.0x	1.30	Oil
S1C-left	3d MI overview actn2/arid1a	HC PL FLUOTAR 20.0x/0.50 DRY	20.0x	0.50	Dry
S1C-right	3d MI zooms actn2/arid1a	HC PL FLUOTAR 20.0x/0.50 DRY	20.0x	0.50	Dry
S2B	P1 zooms ARID1A cKO cre efficiency	ACS APO 63.0x/1.30 OIL	63.0x	1.30	Oil
S2C	P7 zooms ARID1A cKO cre efficiency	ACS APO 63.0x/1.30 OIL	63.0x	1.30	Oil
S5A	YAP immunostaining P7 hearts	UPL XAPO 20X/0.8 DRY	20.0x	0.8	Dry
S6D	Adult icKO cre efficiency	HCX PL APO 63.0x/1.40 OIL	63.0x	1.40	Oil
S7	Overview EdU in IR ARID1A icKO	UPL XAPO 20X/0.8 DRY	20.0x	0.8	Dry

Magn. Magnification; NA Numerical Aperture; IM immersion medium

Supplemental Figures

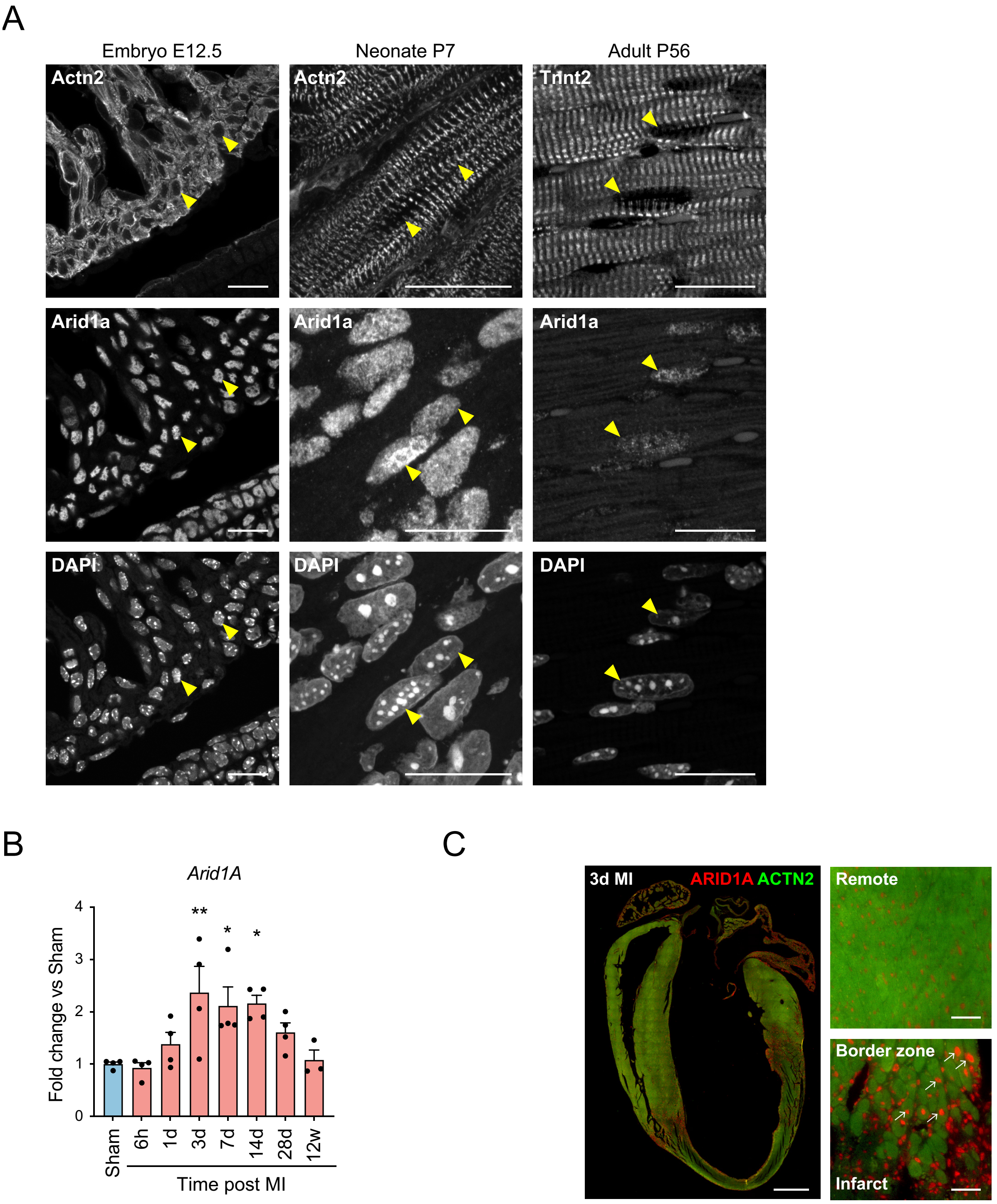

Supplemental Figure 1. *Arid1a* expression in mouse heart after myocardial infarction (MI). A) Representative immunofluorescence staining for cardiomyocytes (ACNT2 or TNNT2, green; top), ARID1A (middle) in embryonic (E12.5), postnatal (P7) and adult mouse (n=3). DAPI marks all nuclei (bottom) **B)** qPCR analysis of *Arid1a* expression in border zone of left ventricular wall in sham and 6 hours (6h), 1 day (1d), 3d (P=0.0069), 1 week (1w; P=0.0329), 2w (P=0.0253), 4w, and 12w after MI (n=4, except 12w: n=3; *, P<0.05, ** P<0.01, compared to sham, one-way ANOVA with Dunnett's test for multiple comparisons). **C)** ARID1A immunostaining in mouse heart 3d after MI shows enhanced expression in border

zone cardiomyocytes (marked by ACTN2, green) compared to cardiomyocytes in remote zone (n=3). Scale bars 1mm (C, left panel), 20 µm (A; C, right panels). Bar graphs show mean with standard error of the mean. Source data are provided as a Source Data file.

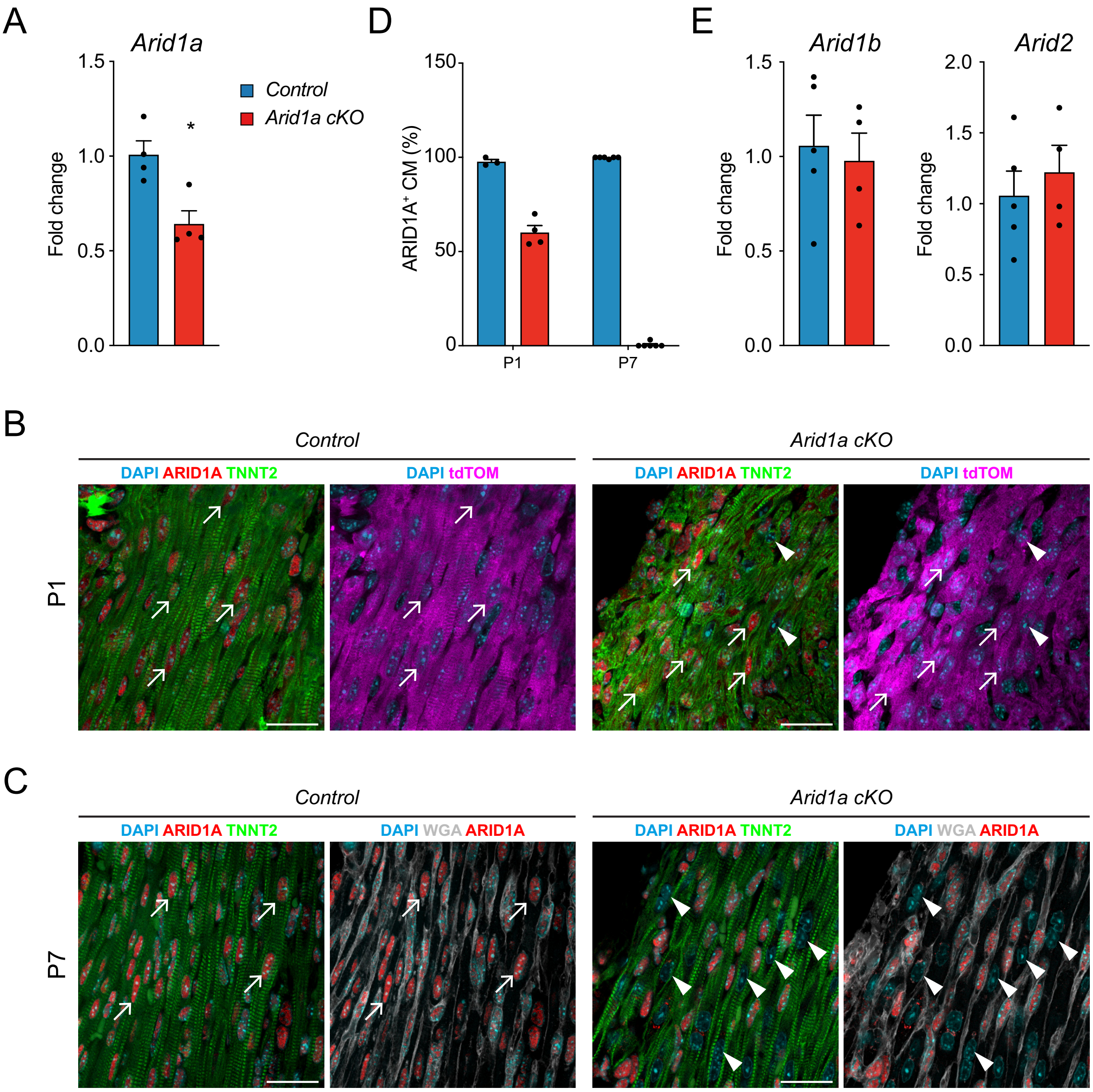

Supplemental Figure 2. Assessment of *Arid1a* expression levels in control and *Arid1a cKO* hearts. A) qPCR for *Arid1a* in ventricular tissue of P7 *control* and *Arid1a cKO* hearts shows a significant reduction of *Arid1a* mRNA levels (P=0.0112), with remaining *Arid1a* expression in *Arid1a cKO* hearts likely reflecting expression in non-cardiomyocytes (n=4; *, P<0.05, two-tailed Student's t-test). **B)** ARID1A expression (red) in P1 *control* and *Arid1a cKO* hearts. tdTomato (tdTom) staining (magenta) was used for marking $R26^{+/tdTom}$ lineage traced cardiomyocytes and reveals that *Cre* was efficiently expressed in TNNT2 expressing cardiomyocytes (green) at this stage. Cardiomyocytes expressing ARID1A are marked with arrows. Arrowheads indicate cardiomyocytes that are negative for ARID1A. **C)** ARID1A expression (red) in P7 control and *Arid1a cKO* hearts. WGA (gray) was used to aid in the identification of non-cardiomyocytes. **D)** Relative counts of ARID1A expressing

cardiomyocytes revealing that at P1, the majority of cardiomyocyte nuclei still express ARID1A in *Arid1a cKO* hearts (n=4; Control, n=3), whereas at P7 (n=6) the expression of ARID1A in cardiomyocytes was lost. **E)** qPCR analysis of *Arid1b* (P=0.7304) and *Arid2* (P=0.5398) reveals no significant difference between controls (n=5) and *Arid1a cKO* (n=4) hearts at P7 (two-tailed Student's t-test). Scale bars 20 µm. Bar graphs show mean with standard error of the mean. Source data are provided as a Source Data file.

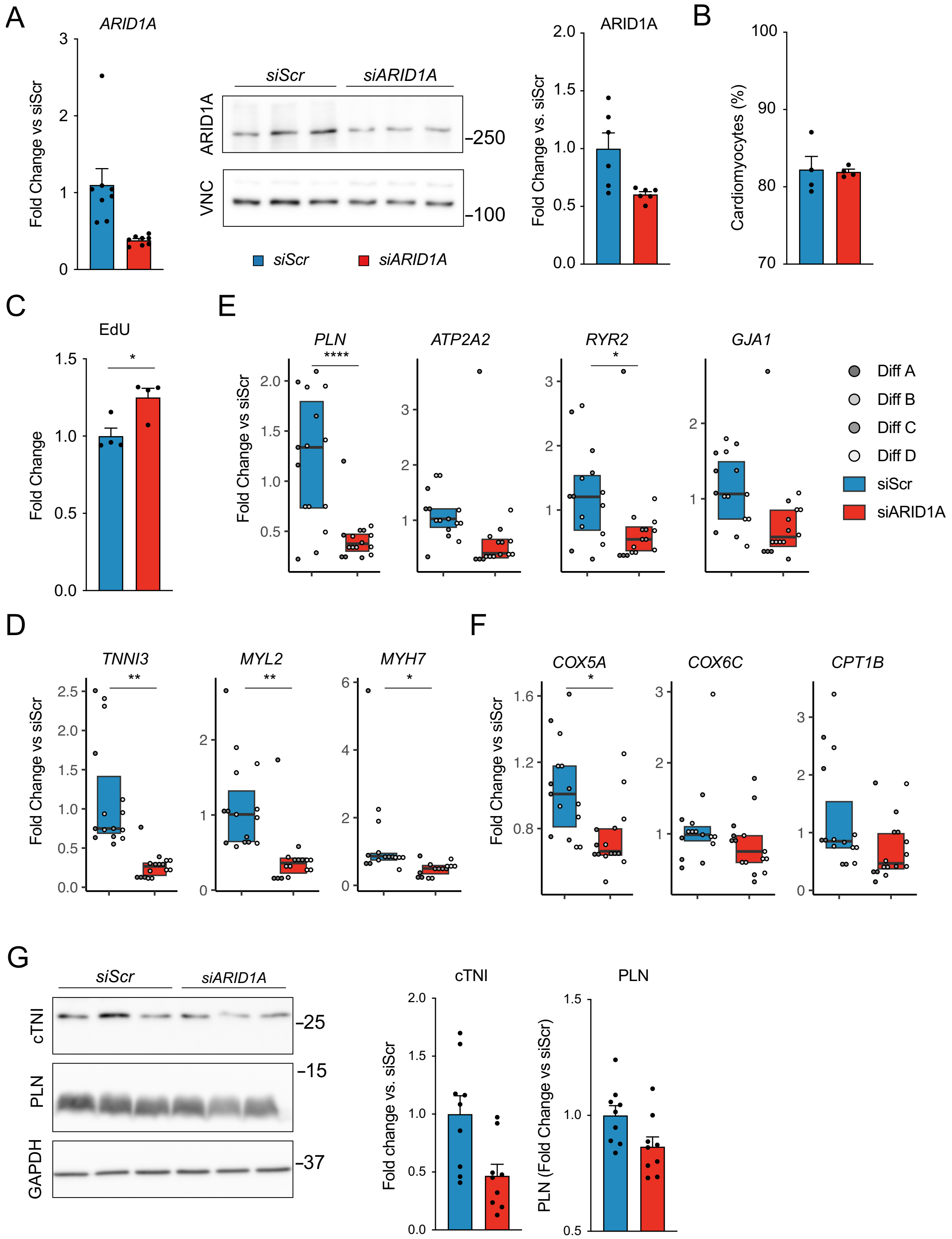

Supplemental Figure 3. Modulation of ARID1A in human iPS-Cardiomyocytes reveals conserved roles in regulation of proliferation and maturation. A) siRNA suppression of *ARID1A* in iPS-CMs validated at mRNA (left, n=2 independent differentiations with 4 technical replicates each) and protein level (middle, right; n2 independent differentiations, with 3 technical replicates each). Molecular weight marker (kDa) is shown at the right. **B)** siRNA treatment does not affect relative number of cardiomyocytes in cultures (n=4). **C)** EdU incorporation in iPS-CMs is significantly induced after suppression of *ARID1A* (P=0.0194; n=4 independent differentiations; * P<0.05, two-tailed Student's t-test). **D)** Expression of mature contractility genes *TNNI3* (P=0.0089), *MYL2* (P=0.0060), and *MYH7* (P=0.0384) was reduced after suppression of *ARID1A*. (n=4 independent differentiations; two-tailed Student's t-test) **E)** Expression of key calcium handling genes *PLN* (P=0.0001), *ATP2A2* (P=0.1147), *RYR2* (P=0.0250), *GJA1* (P=0.0534) after suppression of *ARID1A* (n=4 independent differentiations; two-tailed Student's t-test). **F)** Expression of metabolism genes *COX5A* (P=0.0332), *COX6C*, (P=0.1534) and *CPT1B* (P=0.2267) after suppression of *ARID1A* (n=4 independent differentiations; two-tailed Student's t-test). **G)** Western blot analysis in iPS-CMs after siRNA suppression of *ARID1A* and quantifications of cTNI (P=0.1411) and PLN (P=0.0962; n= 3 independent differentiations; two-tailed Student's t-test). Bar graphs show mean with standard error of the mean. Box plots show inter quartile range with median and dots for each replicate measurement per differentiation. Source data are provided as a Source Data file.

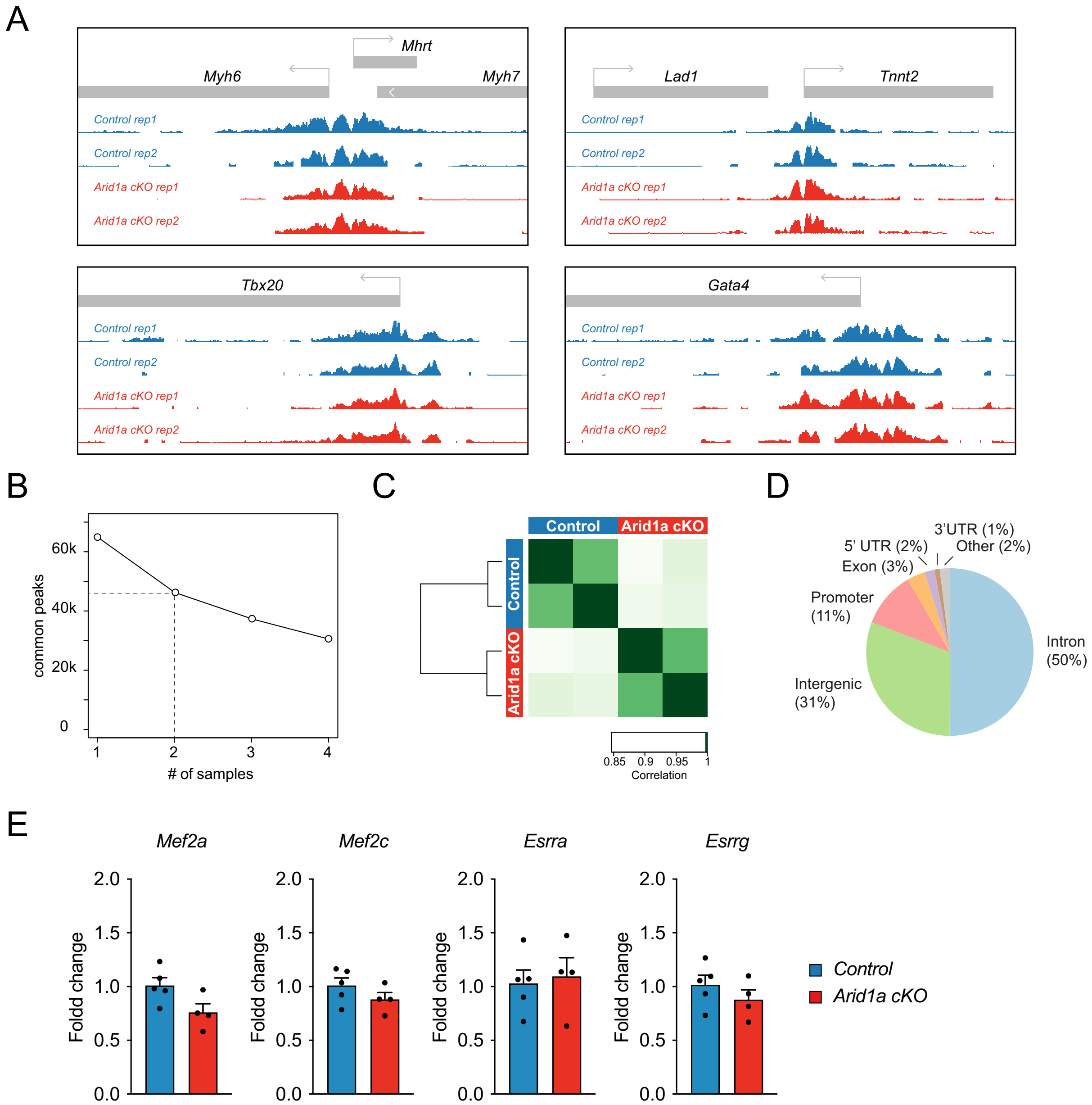

Supplemental Figure 4. H3K27Ac ChIP-Seq results from *Arid1a cKO* and control hearts. **A)** H3K27Ac signal from control and *Arid1a cKO* samples in genomic regions encoding key cardiac genes **B)** Overlap between peaks called from 4 ChIP-Seq samples (2 controls, 2 *Arid1a cKO* hearts). Peaks present in at least 2 samples were used or further analysis. **C)** Correlation heatmap of read counts shows clustering of samples by genotype. **D)** Distribution of peaks over genomic regions. **E)** qPCR analysis of genes associated with enriched transcription factor motifs in Lost peaks indicates that *Mef2*a (P=0.0532), *Mef2c* (P=0.2282), *Esrra* (P=0.7639), and *Esrrg* (P=0.3218) genes are not significantly differentially regulated in *Arid1a cKO* hearts (n=4) compared to controls (n=5; two-tailed Student's t-test). Bar graphs show mean with standard error of the mean. Source data are provided as a Source Data file.

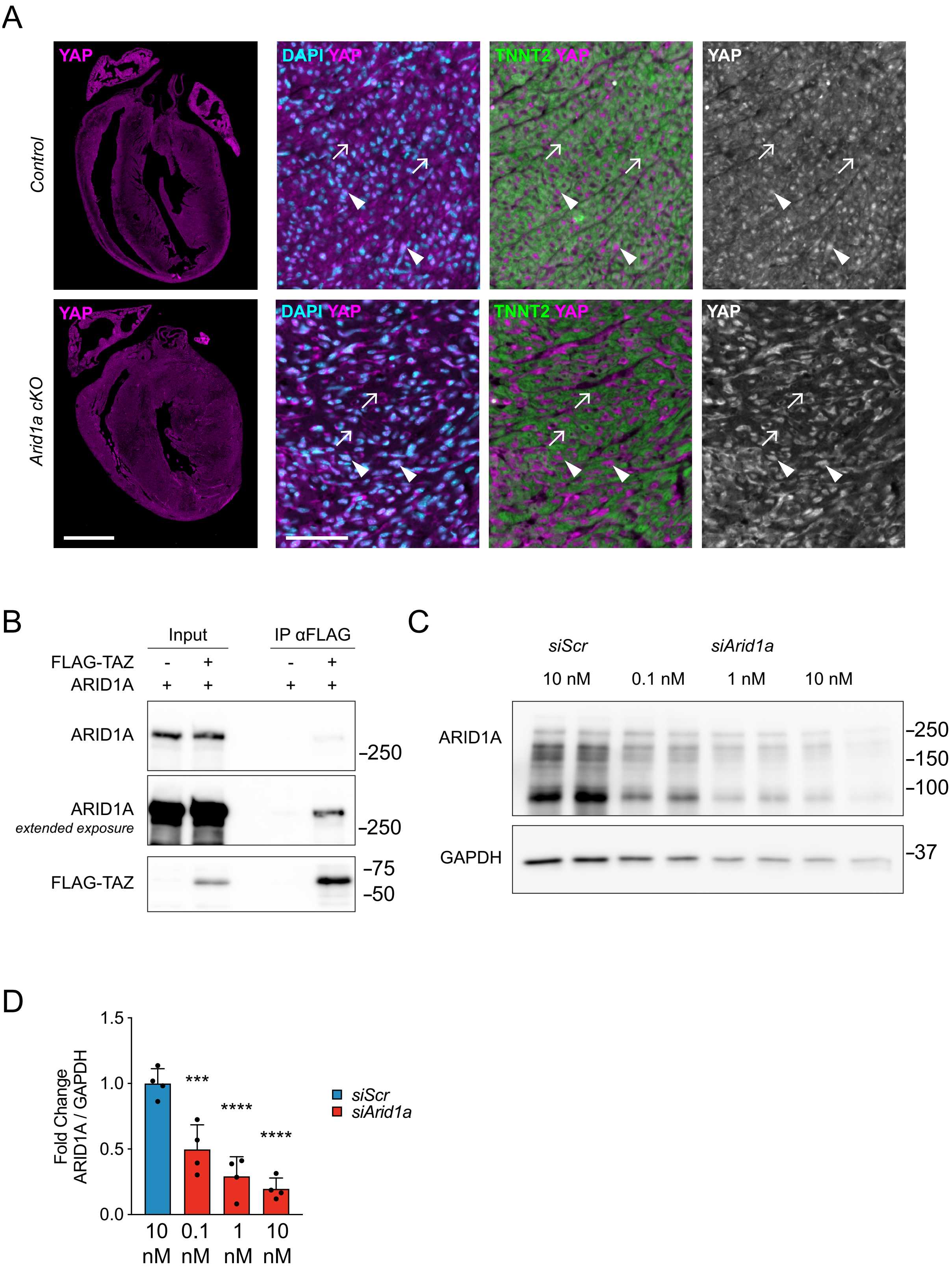

Supplemental Figure 5. ARID1A interacts with YAP and TAZ in a cardiac context. A) Immunofluorescence staining for YAP (magenta) in P7 control (n=5) and *Arid1a cKO* hearts (n=6). Representative examples of left ventricular free wall, arrows indicate CM nuclei, arrows indicate CM cytosolic staining. Co-staining with a nuclear marker (DAPI, cyan) or cytoplasmic cardiomyocyte marker (TNNT2, green) reveals differences in subcellular distribution. Note

relative intensity between CM nuclei (arrowheads) and surrounding cytoplasm (arrows). Scalebars 1mm (overview), 50 µm (other images). **B**) Representative example of co-immunoprecipitation of FLAG-TAZ and ARID1A in H10 cells, showing specific co-precipitation of ARID1A with TAZ (n=3). Molecular weight marker (kDa) is shown at the right. **C**) Western blot analysis of ARID1A after siRNA-mediated knockdown shows dose dependent downregulation of ARID1A relative to GAPDH (right), as quantified in **D**) for 0.1 nM (P=0.0007), 1nM (P<0.0001) and 10 nM (P<0.0001; n=4 technical replicates; ***, P<0.001, ****, P<0.0001, one-way ANOVA with Dunnett's multiple comparison test). Bar graphs show mean with standard error of the mean. Source data are provided as a Source Data file.

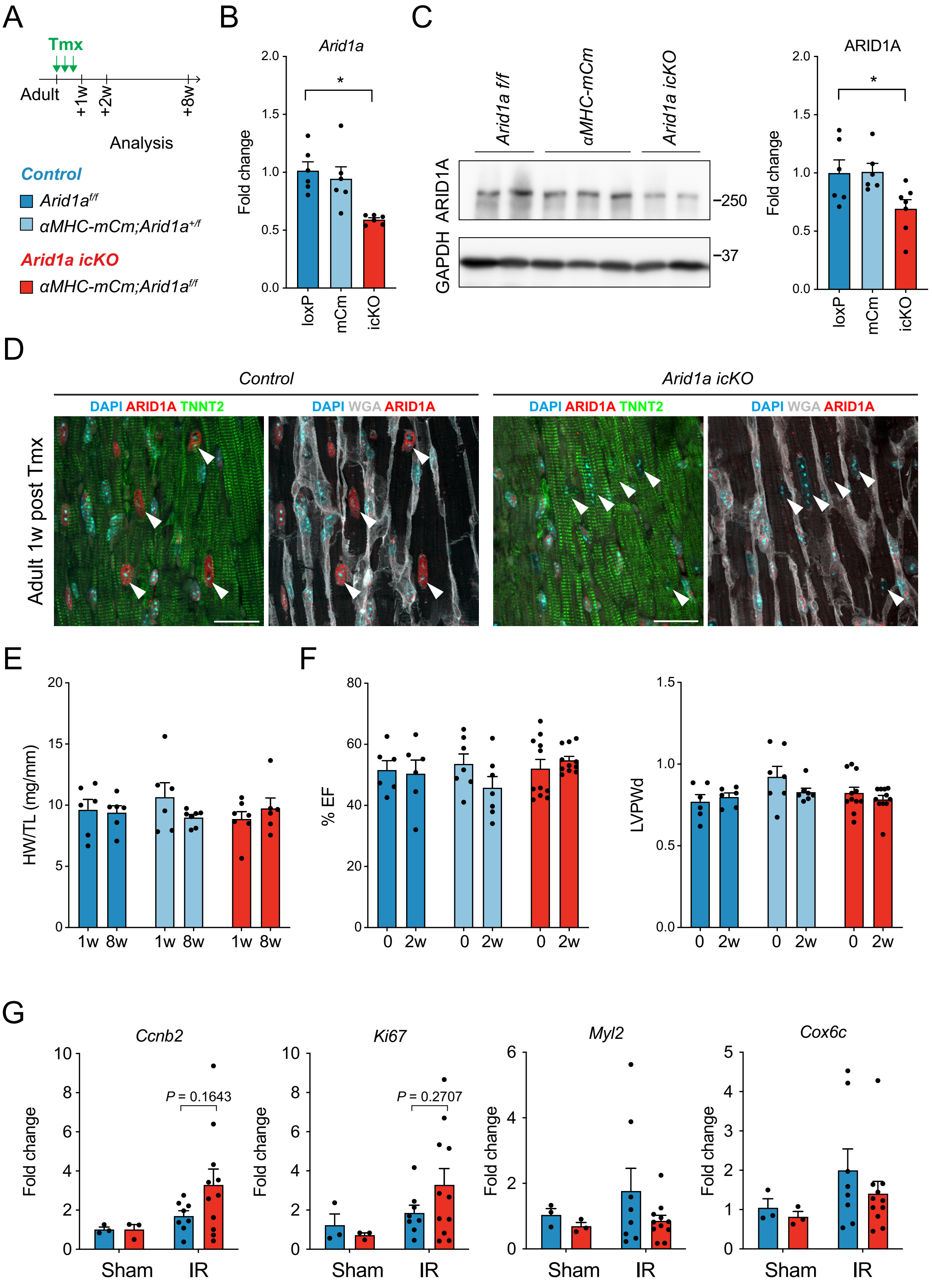

Supplemental Figure 6. Adult *Arid1a icKO* phenotype and validation. A) Schematic showing timeline for Tamoxifen (Tmx) induced conditional deletion of *Arid1a* from adult cardiomyocytes. **B)** *Arid1a* mRNA levels in control hearts (*loxP*, *Arid1a*$^{f/f}$ (blue); *mCm*, *αMHC-mCm;Arid1a*$^{+/f}$ (light-blue)) and *Arid1a icKO* (red; P=0.0241) as measured by qPCR 1

week (w) after tamoxifen (n=6; **, P<0.01, one-way ANOVA with Dunnett's multiple comparisons test, compared to *loxP* control). **C)** Western blot analysis and quantification of ARID1A protein levels in *loxP* (n=6), *mCm* (n=6) and *Arid1a icKO* (n=7; P=0.0453) 1w after tamoxifen (n=6-7; *, P<0.05 versus loxP control, one-way ANOVA with Dunnett's multiple comparisons test, compared to *loxP* control). Molecular weight marker (kDa) is shown at the right. **D)** Immunofluorescence analysis of ARID1A expression in cardiomyocytes 1w after tamoxifen. WGA marks extracellular matrix and together with TNNT2 cardiomyocyte staining is used to identify cardiomyocyte nuclei. Arrowheads mark cardiomyocyte nuclei. Scale bars 25 µm. **E)** Heart weight to tibia length (HW/TL) of control and mutant hearts at 1w and 8w after tamoxifen in *loxP* (n=6), *mCm* (n=6) and *Arid1a icKO* (n=7). **F)** Cardiac functional parameters ejection fraction (EF) and left ventricular posterior wall thickness during diastole (LVPWd) as assessed by echocardiography reveals no functional differences at baseline between *Arid1a icKO* hearts (n=11) and controls (*loxP* (n=6), *mCm* (n=6)) . For legend to colors see A). **G)** qPCR of key cell cycle (Ccnb2, P=0.1643; Ki67, P=2707), contraction (Myl2, P=0.2223) and metabolism genes (Cox6c, P=0.4802), in *Arid1a icKO* and control hearts subjected to Sham (n=3) or IR surgery (Control, n=8; *Arid1a icKO*, n=11). Two-way ANOVA with Šídák's multiple comparisons test, P-values are shown for IR condition. Bar graphs show mean with standard error of the mean. Source data are provided as a Source Data file.

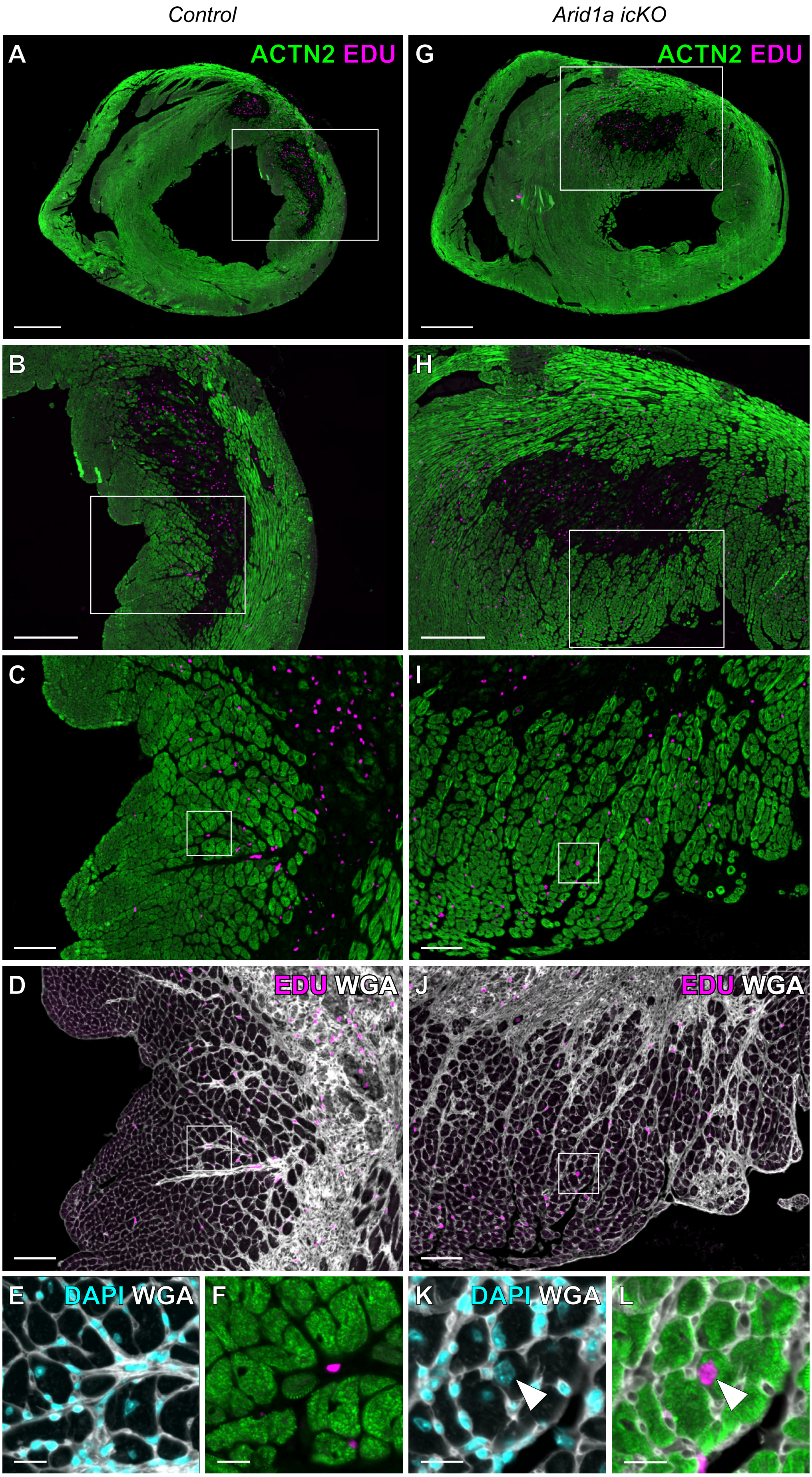
Control
Arid1a icKO
A
ACTN2 EDU
G
ACTN2 EDU
B
H
C
I
D
EDU WGA
J
EDU WGA
E
DAPI WGA
F
K
DAPI WGA
L

Supplemental Figure 7. Overview of EdU labeling in control and Arid1a icKO hearts 7 days after IR. Transversal sections of adult mouse hearts 7 weeks after IR (n=8), stained for DAPI (Cyan, nuclei), WGA (White, extracellular matrix), ACTN2 (Green, cardiomyocytes) and EdU (Magenta). Boxed areas are enlarged in lower panels. Panels C,D and I,J illustrate that most EdU+ cells are ACTN2 negative non-cardiomyocytes. Arrowhead in K,L marks EdU+ cardiomyocyte nucleus. Scale bars 800 µm (A, G), 200 µm (B,H), 100 µm (C,D,I,J), 20 µm (E,F,K,L).
