# Supplementary material for: Cardiomyocyte proliferation is suppressed by ARID1A-mediated YAP inhibition during cardiac maturation
Source: Nat Commun. 2023 Aug 5;14:4716. doi: 10.1038/s41467-023-40203-2 (PMC10404286; doi:10.1038/s41467-023-40203-2)
Supplement: Supplementary file 2 — Description of Additional Supplementary Files [file 41467_2023_40203_MOESM2_ESM.pdf]

Description of Additional Supplementary Files

File Name: Supplementary Data 1
Description: Functional annotation of differentially expressed genes, and differential H3K27Ac ChIP-Seq peaks

File Name: Supplementary Data 2
Description: Peak to gene annotation of differential H3K27Ac ChIP-Seq peaks

File Name: Supplementary Data 3
Description: Transcription factor motif occurrences in differential H3K27Ac ChIP-Seq peaks
