# Supplementary material for: Cardiomyocyte proliferation is suppressed by ARID1A-mediated YAP inhibition during cardiac maturation
Source: Nat Commun. 2023 Aug 5;14:4716. doi: 10.1038/s41467-023-40203-2 (PMC10404286; doi:10.1038/s41467-023-40203-2)
Supplement: Supplementary file 6 — Reporting Summary [file 41467_2023_40203_MOESM6_ESM.pdf]

nature portfolio

Corresponding author(s): CJJ Boogerd, E van Rooij

Last updated by author(s): 05/july/2023

Reporting Summary

Nature Portfolio wishes to improve the reproducibility of the work that we publish. This form provides structure for consistency and transparency in reporting. For further information on Nature Portfolio policies, see our Editorial Policies and the Editorial Policy Checklist.

Statistics

For all statistical analyses, confirm that the following items are present in the figure legend, table legend, main text, or Methods section.

n/a	Confirmed	
☐	☒	The exact sample size (*n*) for each experimental group/condition, given as a discrete number and unit of measurement
☐	☒	A statement on whether measurements were taken from distinct samples or whether the same sample was measured repeatedly
☐	☒	The statistical test(s) used AND whether they are one- or two-sided<br>*Only common tests should be described solely by name; describe more complex techniques in the Methods section.*
☒	☐	A description of all covariates tested
☐	☒	A description of any assumptions or corrections, such as tests of normality and adjustment for multiple comparisons
☐	☒	A full description of the statistical parameters including central tendency (e.g. means) or other basic estimates (e.g. regression coefficient) AND variation (e.g. standard deviation) or associated estimates of uncertainty (e.g. confidence intervals)
☐	☒	For null hypothesis testing, the test statistic (e.g. *F*, *t*, *r*) with confidence intervals, effect sizes, degrees of freedom and *P* value noted<br>*Give P values as exact values whenever suitable.*
☒	☐	For Bayesian analysis, information on the choice of priors and Markov chain Monte Carlo settings
☒	☐	For hierarchical and complex designs, identification of the appropriate level for tests and full reporting of outcomes
☒	☐	Estimates of effect sizes (e.g. Cohen's *d*, Pearson's *r*), indicating how they were calculated

Our web collection on statistics for biologists contains articles on many of the points above.

Software and code

Policy information about availability of computer code

Data collection	Leica Application Suite X (LAS X, version 3.3.0 or newer) was used for image acquisition of confocal microscopy data
Data analysis	DeSeq2 1.24.0 was used for differential expression analysis of RNA-Seq data<br>fGSEA 1.5.2 was used to perform gene set enrichment analysis of RNA-Seq data, using MSigDB collections version 6.2<br>BWA 0.7.17-r1188 was used to align chip-seq reads<br>ChIP-Seq peaks were called using Homer findPeaks (v4.10)<br>Differential peaks were called using Diffbind 2.12.0 and Deseq2 1.24.0<br>Differential peaks were annotated with StringDB v11.0 (http://ww.stringDB.org) gene ontology biological process (GO:BP)<br>Heatmaps for ChIP-Seq data were generated using the computeMatrix (3.3.0.0.0) and plotHeatmap (3.3.0.0.1) functions of the Deeptools package (3.3.0) on usegalaxy.org.<br>Motif discovery was performed using Homer findMotifsGenome package (version 4.11)<br>Fiji (Versions 1.53a or higher) was used for basic image analysis and adjustment of contrast and brightness<br>Qupath v0.4.0 was used for quantifying EdU incorporation in iPS-CM experiments<br>VevoLab version 5.7.1 was used for echocardiography analysis<br>Graphpad Prism (version 9.5.1) was used for statistical analysis

For manuscripts utilizing custom algorithms or software that are central to the research but not yet described in published literature, software must be made available to editors and reviewers. We strongly encourage code deposition in a community repository (e.g. GitHub). See the Nature Portfolio guidelines for submitting code & software for further information.

Data

Policy information about availability of data

All manuscripts must include a data availability statement. This statement should provide the following information, where applicable:

- Accession codes, unique identifiers, or web links for publicly available datasets
- A description of any restrictions on data availability
- For clinical datasets or third party data, please ensure that the statement adheres to our policy

RNA-Seq and ChIP-Seq data are deposited to gene expression omnibus (GEO) with accession number GSE155102. All uncropped gels and numerical values are provided in the Source data file. Mouse mm10 reference genome used in this study is available from http://hgdownload.cse.ucsc.edu/goldenpath/mm10/bigZips/mm10.fa.gz. Source data are provided as a Source Data file.

Research involving human participants, their data, or biological material

Policy information about studies with human participants or human data. See also policy information about sex, gender (identity/presentation), and sexual orientation and race, ethnicity and racism.

Reporting on sex and gender	n/a
Reporting on race, ethnicity, or other socially relevant groupings	n/a
Population characteristics	n/a
Recruitment	n/a
Ethics oversight	n/a

Note that full information on the approval of the study protocol must also be provided in the manuscript.

Field-specific reporting

Please select the one below that is the best fit for your research. If you are not sure, read the appropriate sections before making your selection.

☒ Life sciences ☐ Behavioural & social sciences ☐ Ecological, evolutionary & environmental sciences

For a reference copy of the document with all sections, see nature.com/documents/nr-reporting-summary-flat.pdf

Life sciences study design

All studies must disclose on these points even when the disclosure is negative.

Sample size	Sample size was predetermined for in vivo studies, using power analysis with type I error (alpha) = 0.05, type II error (beta) = 0.2 and effect size of 10%.
Data exclusions	Two Arid1a cKO mutant pups were excluded from the echo analysis at P7, since the quality of the images did not allow for a reliable assesment of wall thickness. The possibility of exclusion of pups due to below threshold quality of echo data was expected, and was taken into account when group sizes were determined prior to the experiment.
Replication	All experiments were performed with a minimal of 3 replicates, and all attempts at replication were succesful, except in the following cases. ChIP-Seq experiments were performed on 2 controls and 2 mutant hearts, in line with recommendations by the encode project f(https://www.encodeproject.org/chip-seq/transcription_factor/). Experiments in iPS-CM were performed in multiple (2 to 4) independent differentiations of the same iPS-cell line, except for the experiments using EHM tissues, where 10 tissues for each condition were cast from the same differentiation of iPS-CM.
Randomization	Mice were allocated to groups based on their genotype. Where possible, littermate controls were used.Randomization was not relevant for in vitro experiments, however, all cells or samples were treated and analyzed in the same manner across conditions.
Blinding	Investigators were blinded to group allocation during data collection (echo) and analysis (echo analysis, proliferation assays, gene expression), except for the echocardiography of P7 pups, where the Arid1a cKO mutants have an apparent phenotype.

Reporting for specific materials, systems and methods

We require information from authors about some types of materials, experimental systems and methods used in many studies. Here, indicate whether each material, system or method listed is relevant to your study. If you are not sure if a list item applies to your research, read the appropriate section before selecting a response.

Materials & experimental systems

n/a	Involved in the study
☐	☒ Antibodies
☐	☒ Eukaryotic cell lines
☒	☐ Palaeontology and archaeology
☐	☒ Animals and other organisms
☒	☐ Clinical data
☒	☐ Dual use research of concern
☒	☐ Plants

Methods

n/a	Involved in the study
☐	☒ ChIP-seq
☒	☐ Flow cytometry
☒	☐ MRI-based neuroimaging

Antibodies

Antibodies used

Antibodies used are listed in the supplemental table 2

Validation

Antibodies were used as directed by the suppliers.
The ARID1A antibody was not confirmed (but predicted) to work with mouse. Data in supplemental figures 2B, 2C, 6B and 6E confirm the sensitivity and specificity of this antibody for mouse in both IF and WB.
ACTN2 - Sigma-Aldrich, A7732 - 1:200 (IF) - Supplier: Species reactivity include human and mouse. Suitable for IF
ARID1A - Abcam, AB182560 - 1:200 (IF) - Supplier: Species reactivity Human. Predicted to work with mouse. Suitable for IF.
ARID1A - Abcam, AB182560 - 1:1000 (WB) - Supplier: Species reactivity Human. Predicted to work with mouse. Suitable for WB
Vinculin - Santa Cruz, sc-25336 - 1:1000 (WB) - Supplier: Species reactivity include human and mouse. Suitable for WB
FLAG - Sigma-Aldrich, F3165 - 1:1000 (WB) - Supplier: Monoclonal ANTI-FLAG® M2 antibody has been used in: immunoblotting, immunoprecipitation
GAPDH - Millipore, MAB374 - 1:5000 (WB) - Supplier: Species reactivity include human and mouse. Suitable for WB
H3K27Ac - Active Motif Cat# 39133 - 1:50 (ChIP) - Supplier: Species reactivity includes mouse. ChIP-grade antibody.
PLN - ThermoFisher, MA3-922 - 1:1000 (WB) - Supplier: Species reactivity include human and mouse. Suitable for WB
tdTomato - SciGen, AB8181 - 1:500 (IF) - Supplier: Suitable for immunohistochemistry - paraffin sections.
TNNI - Abcam, AB47003 - 1:1000 (WB) - Supplier: Species reactivity include human and mouse. Suitable for WB
TNNT2 - Abcam, AB8295 - 1:200 (IF) - Supplier: Species reactivity include human and mouse. Suitable for IF
YAP1 - Cell Signaling, 4912S - 1:1000 (WB) - Supplier: Species reactivity include human and mouse. Suitable for WB
YAP1 p-SER127 - Cell Signaling, 13008P - 1:1000 (WB) - Supplier: Species reactivity include human and mouse. Suitable for WB
YAP1 - Novus anti-Yap, NB110-58358 - 1:200 (IF) - Supplier: Species reactivity include human and mouse. Suitable for IF

Eukaryotic cell lines

Policy information about cell lines and Sex and Gender in Research

Cell line source(s)

HEK293T Lenti-X cells (TaKaRaBio, Cat# 632180)
human iPSC, healthy, White, 31-year-old, male donor (ATCC-BYS0112)
H10 cells (Jahn et al. Journal of Cell Science 1996)

Authentication

Authentication of the cells was not performed

Mycoplasma contamination

human iPSC were routinely monitored for mycoplasma contamination and tested negative.
Hek 293T Lenti-X cells and H10 cells were not tested for mycoplasma contamination

Commonly misidentified lines
(See ICLAC register)

No commonly misidentified cell lines were used in this study.

Animals and other research organisms

Policy information about studies involving animals; ARRIVE guidelines recommended for reporting animal research, and Sex and Gender in Research

Laboratory animals

Mouse lines were maintained on C57B/6J background.
Arid1aloxP (Arid1atm1.1Zhwa/J ; Stock#: 027717)
Rosa26tdTomato (Rosa26tm14(CAG-tdTomato)Hze; Stock#: 007914)
wildtype C57B/6J (Stock#: 000664) mice were obtained from Jackson Laboratories.
αMHC-Cre was obtained from M.D. Schneider (Imperial College, London, UK)
αMHC-mER-Cre-mER was obtained from J.D. Molkentin (University of Cincinnati, OH, USA)

Experiments with were perfomed using pups at P1, P7, or P14.
Experiments with Arid1a icKO mice (aMHC-mER-Cre-mER; Arid1a-loxP) wer performed in adults which were 6-8 weeks old mice at the start of an experiment (i.e. tamoxifen induction or induction of ischemic, injury) and followed for up to 10 weeks.

	Ischemic injury experiments in wild-type mice were performed in adults which were 6-9 weeks old mice at the start of the experiment (induction of ischemic, injury) and followed for up to 8 weeks.
Wild animals	No wild animals were used in this study
Reporting on sex	Animal studies involving pups included males and females.<br>Animal studies involving adult animals (>6w) included males only.
Field-collected samples	No files collected samples were used in this study
Ethics oversight	Animal studies were approved by the animal welfare agency (IvD) of the Royal Dutch Academy of Sciences and Arts (KNAW) and in compliance with national legislation and institutional guidelines.

Note that full information on the approval of the study protocol must also be provided in the manuscript.

Plants

Seed stocks	n/a
Novel plant genotypes	n/a
Authentication	n/a

ChIP-seq

Data deposition

☒ Confirm that both raw and final processed data have been deposited in a public database such as GEO.

☒ Confirm that you have deposited or provided access to graph files (e.g. BED files) for the called peaks.

Data access links<br>*May remain private before publication.*	https://www.ncbi.nlm.nih.gov/geo/query/acc.cgi?acc=GSE155102
Files in database submission	GSM4695455 Input Control rep1<br>GSM4695456 Input Control rep2<br>GSM4695457 Input Arid1a cKO rep1<br>GSM4695458 Input Arid1a cKO rep2<br>GSM4695459 H3K27Ac Control rep1<br>GSM4695460 H3K27Ac Control rep2<br>GSM4695461 H3K27Ac Arid1a cKO rep1<br>GSM4695462 H3K27Ac Arid1a cKO rep2<br>GSE155100_K27Ac_Arid1a_all_peaks_DESEQ2.csv.gz [all peaks]<br>GSE155100_RAW.tar [bedgraph of peaks]
Genome browser session<br>(e.g. UCSC)	n/a

Methodology

Replicates	Two Arid1a cKO hearts plus two littermate control hearts
Sequencing depth	single-end sequencing, 75bp
Antibodies	anti-H3K27Ac (Active Motif, cat# 39133)
Peak calling parameters	peak calling with Homer v4.11 findPeaks -style histone<br># Fold over input required = 4.00<br># Poisson p-value over input required = 1.00e-04<br># size of region used for local filtering = 10000<br># Fold over local region required = 4.00<br># Poisson p-value over local region required = 1.00e-04<br># peak size = 500<br># peaks found using tags on both strands<br># minimum distance between peaks = 1000<br># fragment length = 232<br>Peaks were called independently for each sample, then during diffbind analysis, only peaks that were present in more than one sample were maintained for further analysis.
Data quality	Peaks were called using threshold of more than 4 fold enrichment over input, and more than 4 fold enrichment over local region,

Data quality	with p-values of enrichment over input and over local region below 1.00e-04. During Diffbind analysis, peaks present in at least two samples were maintained, with the false discovery rate threshold set at 0.05.
Software	mapping to reference genome (mm10) with BWA 0.7.17-r1188<br>peak calling with Homer v4.10 findPeaks -style histone<br>Differential peaks were called using Diffbind 2.12.0 and Deseq2 1.24.0<br>Differential peaks were annotated with StringDB v11.0 (http://ww.stringDB.org) gene ontology biological process (GO:BP)<br>Heatmaps for ChIP-Seq data were generated using the computeMatrix (3.3.0.0.0) and plotHeatmap (3.3.0.0.1) functions of the Deeptools package on usegalaxy.org.<br>Motif discovery was performed using Homer findMotifsGenome package (version 4.11)
